# Global, regional, and national temporal trends in mortality and disability-adjusted life years for cardiovascular disease attributable to low temperature during 1990–2019: an age-period-cohort analysis of the global burden of disease 2019 study

**DOI:** 10.3389/fpubh.2024.1414979

**Published:** 2024-10-10

**Authors:** Xiaofei Li, Zeye Liu, Jing Xie, Hua Shao, Ruibing Xia, Yakun Li, Yun Liu, Xiaohan Fan

**Affiliations:** ^1^Department of Cardiology, National Center for Cardiovascular Disease, Fuwai Hospital, Chinese Academy of Medical Sciences & Peking Union Medical College, Beijing, China; ^2^Department of Cardiac Surgery, Peking University People's Hospital, Beijing, China; ^3^College of Basic Medicine and Clinical Pharmacy, China Pharmaceutical University, Nanjing, China; ^4^Department of Pharmacy, Zhongda Hospital, School of Medicine, Southeast University, Nanjing, Jiangsu, China; ^5^Department of Medicine, University Hospital Munich, Ludwig-Maximilians-University Munich (LMU), Munich, Germany; ^6^Laboratory of Experimental Intensive Care and Anesthesiology, Academic Medical Center, Amsterdam, Netherlands; ^7^Department of Information, The First Affiliated Hospital of Nanjing Medical University, Nanjing, China; ^8^Department of Medical Informatics, School of Biomedical Engineering and Informatics, Nanjing Medical University, Nanjing, Jiangsu, China; ^9^Department of Function Test Center, National Center for Cardiovascular Disease, Fuwai Hospital, Chinese Academy of Medical Sciences & Peking Union Medical College, Beijing, China

**Keywords:** low temperature, cardiovascular disease, age-period-cohort analysis, variable risk, global burden disease

## Abstract

**Background:**

Few studies have focused on the region-specific relationship between cardiovascular disease (CVD) and low temperature worldwide.

**Objective:**

We aimed to provide an overview of trends in mortality and disability-adjusted life years (DALYs) for CVD and its subtypes attributable to low temperature over the past 30 years in 204 countries and regions, along with the associations of these trends with age, period, and birth cohorts.

**Methods:**

Data on the estimated burden of CVDs (including ischemic heart disease, hypertensive heart disease, and stroke) attributable to low temperature were obtained from the Global Burden of Disease Study 2019. We utilized an age-period-cohort model to estimate overall annual percentage changes in mortality (net drifts), annual percentage changes from 15 ~ 19 to 81 ~ 85 years (local drifts), and period and cohort relative risk (period/cohort effects) between 1990 and 2019.

**Results:**

Among noncommunicable diseases, CVDs had the highest mortality rate and DALY loss attributable to low temperature worldwide and has increased from 65.7 to 67.3%, which is mainly attributed to the increase in East Asia and Pacific region. In terms of the level of economic and social development, an inverted U-shape was found in the age-standardized mortality rates (ASMR) due to low-temperature across different sociodemographic indices (SDI) regions. Both high CVD mortality (19.45, 95% CI [14.54, 24.17%]) and a decreasing mortality rate related to low temperature (from 1990 to 2019, net drift, −3.25% [−3.76, 2.73%] per year) was found in high SDI countries or territories, with opposite outcome found in low SDIs regions. The older adults (70+) and men share the highest rate of CVD ASMR and DALY attributed to low temperature across all regions, especially in North America and Europe and Central Asia.

**Conclusion:**

Mortality and DALY loss from CVD attributable to low temperature showed an overall decreasing trend globally except for East Asia and Pacific region. SDI, sex, age and geographic location contributed to the diversity of the CVD disease burden associated with low temperature worldwide. More attention should be given to the older adults, men, and low SDI regions.

## Introduction

Cardiovascular disease is a leading cause of death and morbidity globally (1). It is known that heart attack can be triggered by short-term exposure to environmental factors such as non-optimal ambient temperature. Previous studies have found U- or J-shaped associations between temperature and mortality risks from cardiovascular causes (2–4). However, low temperature has a greater overall effect on cardiovascular mortality than does high temperature (5). In many temperate countries, mortality and hospitalization rates for coronary heart disease and stroke are greater in winter than in summer, and mortality rates for certain diseases can reach as high as 70% in winter as in summer (6). Globally, in the past 30 years from 1990 to 2019, cardiovascular disease (CVD) deaths attributable to cold temperatures increased globally from 812,230 (652,280, 975,870) to 1,104,200 (897,780, 1,326,970), representing a 35.9% increase (7). Due to the wide heterogeneity in climate, socioeconomic development and health all over the world, there may be spatial heterogeneity in temperature-attributable mortality across different countries and regions, and this significant variations have undoubtedly become a major challenge for human health (7). Moreover, in the present context of global conflicts (Russo-Ukrainian war, Israeli-Palestinian conflict, *et al*), energy crisis and energy mix reform (8), and climate change, the health effects of low temperature should receive more attention.

A study explored the relationship between low temperature and the burden of noncommunicable diseases (NCDs) on a global scale (9). Some national-level studies of low temperature and CVDs have also been conducted (10, 11). However, global burden analyses of CVDs attributable to low temperature are lacking. The above studies did not distinguish between the relative contributions of age, period, or cohort to mortality or between specific disease types. Analysis of mortality and disability-adjusted life year (DALY) trends, particularly their relationships with age, period, and cohort effects, has the potential to evaluate the success of the current health care system versus its inadequacy in clarifying the direction of improvement (12, 13). These analyses provide an important basis for developing disease prevention and control strategies specific to each country and region. In-depth analysis of temporal trends in mortality and DALYs for all countries is necessary to obtain relevant information on disease prevalence and to prioritize health care resources. The “low temperature” factor is strongly influenced by geography and the climatic environment. Therefore, the analysis of disease burden should be conducted in conjunction with sociodemographic index (SDI) levels (14) and geographic information.

To our knowledge, this is the first study to use Global Burden of Disease (GBD) Study 2019 data and age-period-cohort (APC) models to explore trends in mortality, DALYs and their subtypes (ischemic heart disease, hypertensive heart disease, and stroke) attributable to low temperature in 204 countries and regions from 1990 to 2019. This manuscript was produced as part of the GBD Collaborators Network in accordance with the GBD protocol.

## Materials and methods

### Data and definitions

The GBD Study uses deidentified data; therefore, a waiver of informed consent was obtained from the University of Washington Institutional Review Board. The GBD 2019 edition provides the most recent estimates of descriptive epidemiologic data for 369 diseases and injuries in 204 countries and regions over 30 years from 1990 to 2019. Each of these events (morbidity, mortality, health loss, etc.) is attributed to a unique and mutually exclusive underlying cause (including all types of diseases and injuries) (7). The GBD Study assigns an SDI to each country, which is a composite of *per capita* income, average years of schooling, and fertility rate for women under 25 years of age. The SDI ranges from 0 to 1, with higher values indicating higher socioeconomic levels. All countries are divided into five categories according to SDI values from highest to lowest. In addition, the GBD Study divides the globe into seven super regions and 21 regions (World Bank criteria). We analyzed changes in disease burden globally and in each region separately and compared the results with those of the SDI-based analysis to explore the impact of the physical environment and the level of economic and social development on the burden of CVD and its subtypes attributable to low temperature. The term “low temperature” in the study refers to temperatures below the theoretical minimum risk exposure level (TMREL) for the given location and year in the GBD Study, and the daily averages of temperature for each location were obtained from the European Center for Medium-Range Weather Forecasts. In the GBD studies, TMREL means the temperature associated with the lowest mortality risk for all included causes combined, was estimated for a given location and year (15). Given varying TMREL for different regions and countries, years, and diseases, the GBD study 2019 employed both spatially and temporally varying to estimate TMREL and are not using a globally uniform TMREL (16, 17). Exposure to non-optimal low temperature is defined as the same-day exposure to ambient temperature that is colder than the temperature associated with the minimum mortality risk. Specific information on the data sources can be obtained from the official GBD website. The data used in the study were obtained directly from the GBD database. The reliability of the data has been demonstrated in previous studies (9, 18).

### Overall temporal trend analysis of mortality and DALYs

Temporal trends in mortality or DALYs were assessed by age-standardized rates (ASR) and the relative percentage change between 1990 and 2019. ASR were calculated using global age-standardized population data from the GBD 2019 (19). The population was divided into six age groups (15–39, 40–49, 50–59, 60–69, 70–79, and 80+), and the proportion of deaths or DALYs in each age group was calculated. Additionally, due to the small number of CVD deaths among individuals aged under 15 in many countries and territories, it is challenging to achieve statistical significance when conducting age-period-cohort analysis for these age groups. Therefore, we only included individuals between the ages of 15 and 80+ years in our APC model analysis. The detailed methods for calculating mortality and DALYs are available in several publications (14, 18).

### Age-period-cohort modeling and analysis

In this study, the APC model was used to analyze potential trends in mortality and DALYs by age, period, and birth cohort. The APC model aims to reveal the contribution of age-related biological factors as well as technological and social factors to disease trends, which is difficult to achieve with traditional epidemiological analysis methods (20). APC modeling has been used in the epidemiological analysis of certain chronic diseases (e.g., CVDs and tumors) (20, 21). The age effect refers to the change in the probability of occurrence of an outcome event with changes in age factors, reflecting the effect of demographic changes on the outcome event. The period effect refers to the impact on the probability of outcome events because different studies were carried out in different years, reflecting the effect of social, economic, and cultural factors in a certain period, such as the effect of public health interventions. The cohort effect refers to the impact on the probability of outcome events due to different birth cohorts with different exposures to certain factors, such as differences in educational attainment, nutritional status, and other factors among people in different birth cohorts. Cohort effects are an important part of the APC model and play a prominent role in cancer and chronic disease occurrence or mortality trends (20). Typically, APC models fit log-linear Poisson models on Lexis plots of observed rates and quantify the additive effects of age, period, and birth cohorts. Estimating the statistically independent effects of age, period, and cohort is not possible due to the perfect linearity of the relationships between the three variables (birth cohort = period–age), i.e., the so-called identification problem. The present study circumvents this problem by generating estimable APC parameters and functions without imposing arbitrary constraints on the model parameters. APC methodology was carried out through a freely-available user-friendly web tool^1^ and Software R, version 4.2.2, and epiR package, version 2.0.59, were used to perform the analysis.

In the present study, the APC model input data included population data and mortality and DALY estimates for CVD patients for each country/region from 1990 to 2019. Age and period intervals must be equal in a typical APC model. However, GBD data are generated in an unequal interval format (five-year age groups with annual data). Therefore, mortality, DALY, and population data were divided into consecutive 5-year periods from 1990 to 2019 (1990–1994 [1992], 1995–1999 [1997] … 2015–2019 [2017]), and the age intervals for each period were 15–19 years, 20–24 years … 75–79 years, and 80 years and older. The sample consisted of 19 consecutive cohorts, including those born between 1908 and 1912 (median, 1910) to between 1998 and 2002 (median, 2000), while the cohort born between 1953 and 1957 (median, 1955) served as the reference group. The fitted APC model estimated the overall time trends in mortality and DALYs by combining age, period, and cohort effects and presented them using net drift (annual percentage change obtained after accounting for the above effects). The net drift was jointly determined by the trend components attributable to calendar time and trend components attributable to the continuous cohort. In addition, to reflect the trend in birth cohort effects, the APC model estimated the time trend in mortality within each age group, expressed as the annual percentage change in age-specific mortality (i.e., the local drift in mortality in percent per year). Relative risk was calculated as the ratio of age-specific rates within each period (cohort) relative to the reference period (cohort). Both period (cohort) rate curves contained the entire value of the net drift. The choice of the reference period (cohort) was arbitrary and did not affect the interpretation of the results. The significance of the annual percentage change trend was tested by the Wald chi-square test. Statistical tests were two-way; *p* < 0.05 was considered to indicate statistical significance. All analyses were performed in R (version 4.2.2).

## Results

### Global burden of CVDs attributable to low temperature between 1990 and 2019

**Figure 1:**
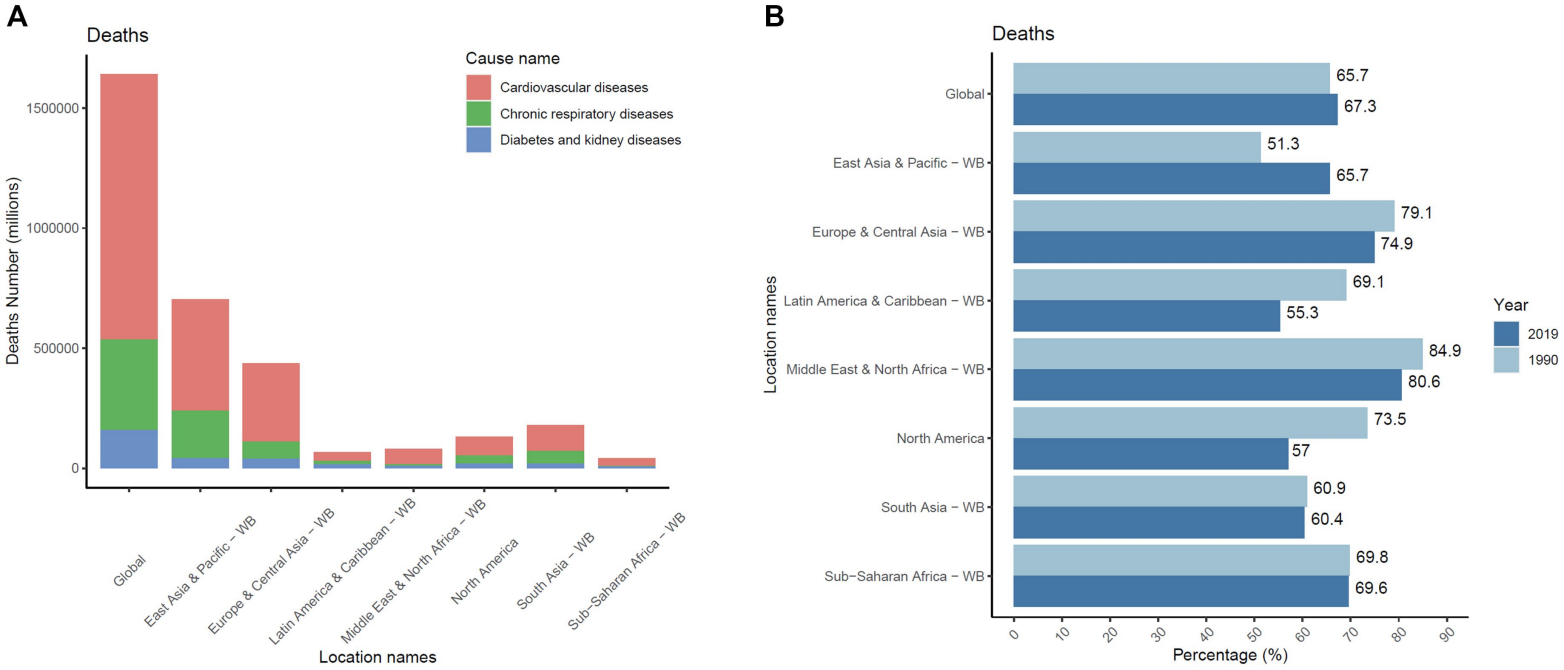
CVD is the leading cause of global mortality attributed to low temperatures from NCDs in the population worldwide. (A) The number of deaths from NCDs due to low temperature in 2019 and cardiovascular diseases accounted for the largest population. (B) Changes in the proportions of CVD-related deaths across different regions from 1990 to 2019. CVD, cardiovascular disease; NCD, noncommunicable disease.

In 2019, the number of NCD deaths attributable to cold temperatures was estimated to be 1.64 million worldwide (95% uncertainty interval [UI] = 1.41–1.90 million; Figure 1A). CVDs accounted for the largest share of these deaths, at more than 67% (1.10 million [0.90–1.33 million]). Globally, the share of CVD-related deaths attributable to cold temperature among NCDs increased from 65.7 to 67.3% between 1990 and 2019 (Figure 1B). An increase was observed only in East Asia and the Pacific, with a net increase of 14.4% (from 51.3 to 65.7%). A decreasing trend was found in all of the areas except East Asia and the Pacific, with the fastest decreases occurring in North America, Latin America and the Caribbean (net decreases of 16.5 and 13.8%, respectively). A slight decrease was observed in South Asia and sub-Saharan Africa. The change in DALYs was similar to the change in mortality (Supplementary Figure S1), and South Asia, the Middle East and North Africa (MENA), and the East Asia and Pacific regions were the three regions with the greatest increases in DALYs (percentage change, 118.42% [76.00, 218.00], 71.57% [47.00, 98.00], and 40.53% [19.00, 66.00], respectively).

### Global and regional trends in CVD-related deaths and DALY losses attributable to low temperature from 1990 to 2019

**Figure 2:**
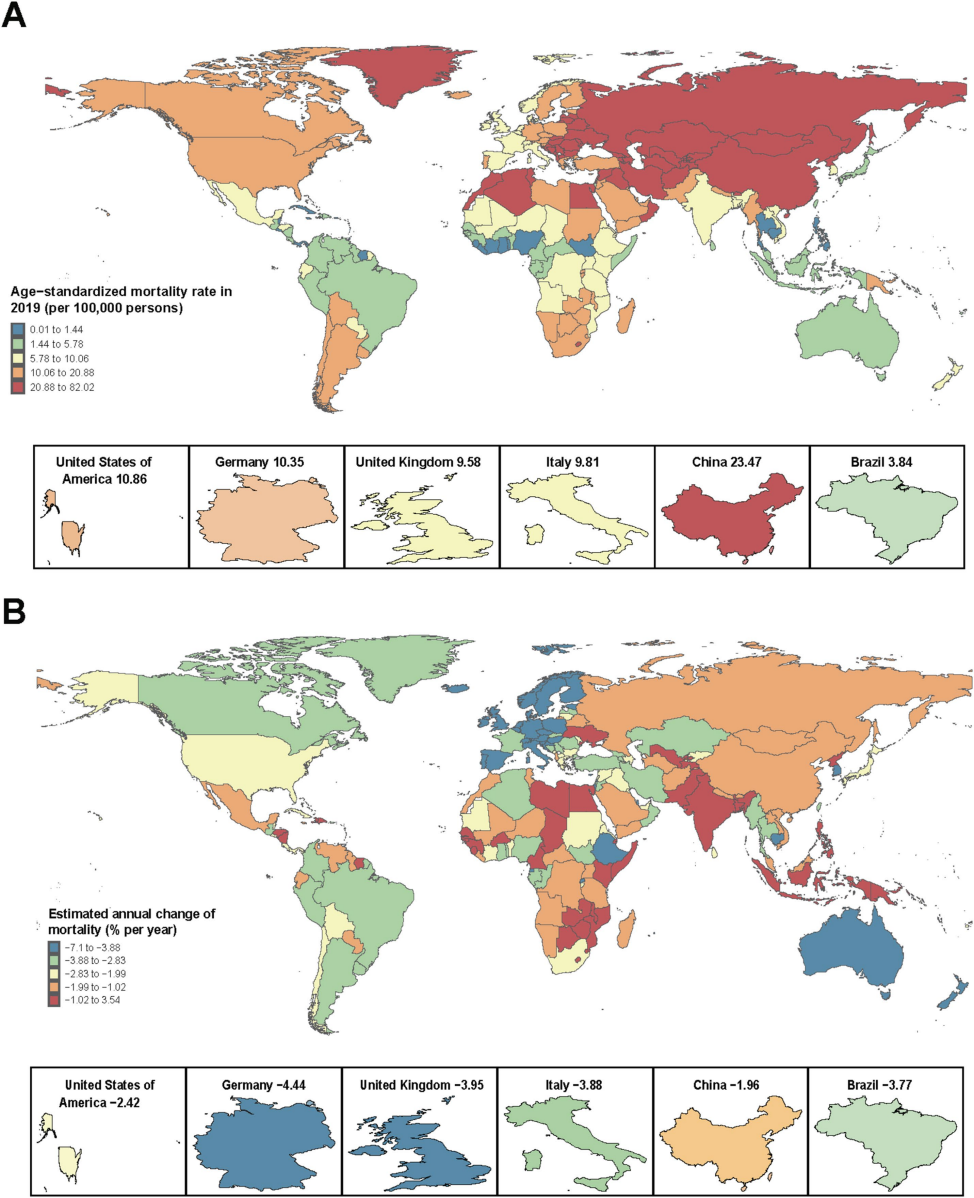
The all-age mortality in 2019 (A) and net drift of mortality attributed to low temperature during 1990–2019 (B) for CVDs in 204 countries and territories. (A) World map of all-age mortality for CVD patients. In 2019, the global all-age mortality was 14.27 (95% UI 11.6–17.15) per 100,000 people. (B) World map of the net drifts for CVD mortality, i.e., estimated annual percentage change in mortality from the age-period-cohort model. Net drift captures components of the trends attributable to calendar time and successive birth cohorts. The global net drift of CVD mortality was −2.19 [95% CI 2.34 to −2.05]. CVD, cardiovascular disease.

Tables 1, 2 and Figure 2 show the net drift in population, total deaths, all-age mortality, age-standardized mortality rates (ASMR), and mortality number. Over the past 30 years, the number of CVD deaths attributable to low temperature increased from 812 thousand (652–976 thousand) to 1,104 thousand (898–1,326 thousand) worldwide, representing an increase of 35.9%. South Asia, the Middle East, North Africa, and East Asia and the Pacific had the greatest changes in percentage of CVD deaths. However, Europe, Central Asia, and North America presented negative or slight increases in the percentage of CVD deaths attributed to low temperature (Table 1). The age-standardized mortality rates attributed to low temperature showed a decreasing trend, and the net drift in mortality for the entire population ranged from −2.87 (−3.01, −2.74) in Latin America and the Caribbean to −0.72 (−0.89, −0.55) in South Asia per year from 1990 to 2019. The disease burden profile shows different trends across regions, and interregional differences may be increasing. For example, Latin America and the Caribbean had a smaller disease burden and showed significant improvement in subsequent years. The MENA region and the European and Central Asian regions had relatively greater disease burdens attributed to low temperature but showed a stronger decreasing trend. Despite the smaller disease burden in South Asia, improvement was also slower. In terms of the level of economic and social development, an inverted U-shape was found in the ASMR in 2019 (Table 2), with the lowest in high-SDI countries (9.0 [6.85–11.15] per 100,000) and low-SDI countries (9.68 [6.4, 12.77] per 100,000) and the highest in high-moderate-SDI countries (9.68 [6.4, 12.77] per 100,000). A decreasing trend was found in the net drift in mortality across the five SDI groups, ranging from −3.25% (95% CI: −3.76, −2.73) per year in high-SDI countries to −1.05% (95% CI: −1.15, −0.94) in low- and medium-SDI countries. Thus, countries with higher SDIs tend to have lower mortality rates, with a more pronounced improvement trend. Supplementary Table S1 and Supplementary Figure S2 show age-standardized rates and net drift for DALYs, with trends essentially identical to mortality trends.

**Table 1:** Trends in CVD mortality attributed to low temperature across different regions, 1990–2019.

	Global		East Asia and Pacific		Europe and Central Asia		Latin America and Caribbean		Middle East and North Africa		North America		South Asia		Sub-Saharan Africa	
	1990	2019	1990	2019	1990	2019	1990	2019	1990	2019	1990	2019	1990	2019	1990	2019
Deaths																
Number, n × 1,000	812.23 (652.28,975.87)	1104.2 (897.78,1326.97)	263.65 (218.18,312.99)	462.45 (375.43,560.28)	349.46 (251.41,458.55)	327.73 (242.1,427.99)	29.66 (23.57,36.23)	37.4 (29.01,46.03)	32.72 (23.88,41.63)	64.37 (46.62,81.77)	72.28 (55.74,89.6)	74.32 (58.23,90.55)	45.75 (22.19,68.01)	107.9 (58.69,156.18)	17.63 (12.54,22.92)	28.45 (19.79,37.57)
Percentage of global, %	100	100	32.46	41.88	43.02	29.68	3.65	3.39	4.03	5.83	8.9	6.73	5.63	9.77	2.17	2.58
Percent change in deaths 1990–2019, %	35.95 (25.0,48.0)		75.4 (50.0,105.0)		−6.22 (−12.0,1.0)		26.1 (16.0,37.0)		96.72 (73.0,122.0)		2.82 (−3.0,8.0)		135.88 (92.0,241.0)		61.34 (45.0,82.0)	
All-age mortality rate																
Rate per 100,000	15.18 (12.19,18.24)	14.27 (11.6,17.15)	14.06 (11.64,16.7)	19.65 (15.95,23.8)	40.95 (29.46,53.74)	35.63 (26.32,46.53)	6.76 (5.37,8.26)	5.76 (4.47,7.09)	12.62 (9.21,16.06)	14.04 (10.17,17.83)	25.73 (19.84,31.9)	20.39 (15.97,24.84)	4.06 (1.97,6.04)	5.78 (3.15,8.37)	3.45 (2.45,4.48)	2.54 (1.77,3.36)
Percent change in rate 1990–2019, %	−6 (−14,2)		39.7 (19,63)		−12.99 (−19, −6)		−14.81 (−22, −7)		11.19 (−2,26)		−20.77 (−25, −16)		42.41 (16,106)		−26.24 (−34, −17)	
Age-standardized mortality rate, ASMR																
Rate per 100,000	24.47 (19.42,29.51)	14.38 (11.72,17.29)	24.67 (20.55,28.98)	16.19 (13.08,19.56)	33.92 (24.4,44.6)	18.86 (13.9,24.76)	12.89 (10.25,15.82)	5.81 (4.51,7.15)	32.66 (24.06,41.38)	24.55 (17.98,30.99)	19.8 (15.29,24.53)	10.8 (8.51,13.09)	9.95 (4.91,14.89)	8.42 (4.59,12.19)	9.66 (6.95,12.52)	7.55 (5.3,9.93)
Percent change in rate 1990–2019, %	−41.25 (−46, −36)		−34.37 (−44, −24)		−44.39 (−48, −4)		−54.92 (−58, −51)		−24.84 (−33, −16)		−45.47 (−48, −43)		−15.39 (−3,21)		−21.9 (−3, −12)	
APC model estimates*																
Net drift of mortality y, % per year	−2.19 (−2.34, −2.05)		−2.04 (−2.23, −1.85)		−2.58 (−2.98, −2.17)		−2.87 (−3.01, −2.74)		−2.18 (−2.26, −2.1)		−2.5 (−2.91, −2.09)		−0.72 (−0.89, −0.55)		−1.97 (−2.07, −1.86)	

*Age range for APC model, 15–80 + .

**Table 2:** Trends in CVD mortality attributed to low temperature across sociodemographic index quintiles, 1990–2019.

	Global		High SDI (*N* = 41)		High-middle SDI (*N* = 41)		Middle SDI (*N* = 40)		Low-middle SDI (*N* = 41)		Low SDI (*N* = 41)	
	1990	2019	1990	2019	1990	2019	1990	2019	1990	2019	1990	2019
Deaths												
Number, n × 1,000	812.23 (652.28,975.87)	1104.2 (897.78,1326.97)	224.89 (164.86,286.66)	197.14 (147.31,244.95)	320.08 (247.57,405.27)	389.37 (305.08,491.98)	183.56 (152.83,215.57)	345.36 (280.94,410.11)	61.21 (41.94,79.03)	129.82 (93.61,165.47)	22.28 (14.84,29.32)	42.23 (27.81,55.54)
Percentage of global, %	100.0	100.0	27.69	17.85	39.41	35.26	22.6	31.28	7.54	11.76	2.74	3.82
Percent change in deaths 1990–2019, %	35.95 (25.0,48.0)		−12.34 (−484.28, −4.84)		21.65 (11.71,31.19)		88.14 (65.05,114.63)		112.1 (88.17,154.2)		89.56 (61.87,135.15)	
All-age mortality												
Rate per 100,000	15.18 (12.19,18.24)	14.27 (11.6,17.15)	27.36 (20.06,34.87)	19.45 (14.54,24.17)	27.82 (21.52,35.23)	27.22 (21.33,34.39)	10.69 (8.9,12.56)	14.41 (11.72,17.11)	5.42 (3.71,7)	7.36 (5.31,9.38)	4.22 (2.81,5.55)	3.74 (2.46,4.92)
Percent change in rate 1990–2019, %	−6 (−13.5, 2.45)		−28.89 (−34.44, −22.81)		−2.16 (−10.16,5.51)		34.77 (18.23,53.75)		35.83 (20.5,62.79)		−11.3 (−24.25,10.03)	
Age-standardized mortality rate, ASMR												
Rate per 100,000	24.47 (19.42,29.51)	14.38 (11.72,17.29)	21.6 (15.86,27.56)	9 (6.85,11.15)	35.52 (27.59,44.98)	19.82 (15.52,25.04)	22.67 (18.91,26.58)	16.52 (13.49,19.58)	12.77 (8.88,16.43)	10.9 (7.9,13.83)	11.51 (7.7,15.15)	9.68 (6.4,12.77)
Percent change in rate 1990–2019, %	−41.25 (−45.88, −35.82)		−58.31 (−61.15, −54.86)		−44.2 (−48.47, −39.93)		−27.16 (−35.63, −17.56)		−14.66 (−23.74,0.9)		−15.91 (−27.55,3.45)	
APC model estimates*												
Net drift of mortality y, % per year	−2.19 (−2.34, −2.05)		−3.25 (−3.76, −2.73)		−2.83 (−3.13, −2.53)		−1.82 (−1.92, −1.71)		−1.05 (−1.15, −0.94)		−1.13 (−1.21, −1.04)	

*Age range for APC model, 15–80 + .

### National death and DALY loss trends in patients with CVD attributable to low temperature from 1990 to 2019

There were 105 countries with at least 500 deaths in 2019 out of 204 countries and regions worldwide. China (392,404 [95% UI: 31400–484,000]), Russia (86,634 [47053–142,993]), India (73,282 [36,147–110,735]), the United States (66,378 [53,143–80,904]), and Ukraine (34,007 [21,812–49,277]) reported the greatest number of deaths, accounting for 59.1% of global deaths. In addition, 21 countries showed a rising trend (net drift ≥0.0%) or stagnation and decline (≥ − 0.5%) in mortality. Of these, seven countries and regions showed a clear upward mortality trend (net drift ≥1.0% per year). Every 1% increase in net drift per year implies a 10, 18, and 26% increase in mortality in the populations of these countries and regions in the next 10, 20, and 30 years, respectively. The Philippines (3.54%, [95% CI: 2.75, 4.34]), Zimbabwe (2.24%, [95% CI: 1.36, 3.12]), Kiribati (2.21%, [95% CI: −12.3, 19.13]), Lesotho (2.09%, [95% CI: 0.85, 3.35]), and Mozambique (1.83%, [95% CI: 1.04, 2.62]) were the five fastest-rising countries. Most of these countries and regions have low or medium-low SDI levels. In terms of DALY losses in 2019, similar trends were found, and China (7,003,989 [5,554,315–8,713,804]), India (1,7,575,580 [859,443–2,665,342]), Russia (1,471,259 [788,354–2,479,253]), the United States (1,023,066 [822,912–1,230,926]), and Ukraine (585,039 [374,937–857,500]) experienced the greatest losses. As the only region with increased CVD deaths attributed to low temperature, East Asia and the Pacific accounted for 41.88% of the global CVD deaths, of which 84.8% occurred in China. For DALYs, in South Asia, India accounted for 66.8% of the overall DALYs; in the MENA region, Egypt, Iran, Morocco and Iraq accounted for 66.8% of the overall DALYs; and in East Asia and the Pacific, China accounted for 85.7% of the overall DALYs. Since all of these countries belong to the low-middle and middle SDI categories, the number of deaths and DALY losses are influenced by various factors, such as the level of economic development, geographic location, and population. The distributions and changes in ASMR and DALYs in 204 countries with different SDI levels are shown in Supplementary Tables S2, S3, respectively.

### Temporal trends in the population distribution of CVD deaths and DALYs among different age groups

**Figure 3:**
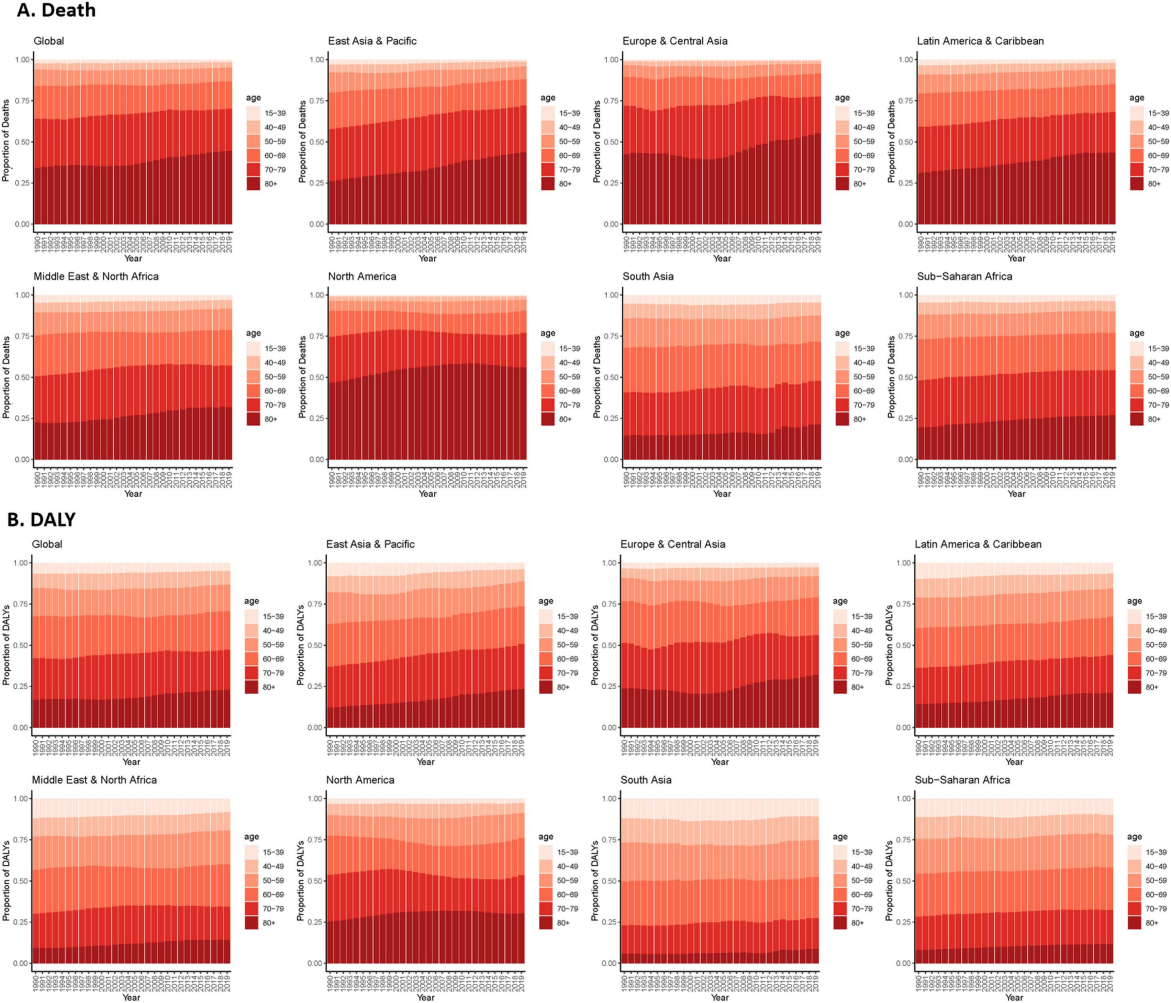
Age distribution of CVD deaths **(A)** and DALYs **(B)** attributed to low temperature. CVD, cardiovascular disease; DALY, disability-adjusted life years.

Figure 3 shows the temporal trends in the age distribution of deaths and DALYs in patients with CVD attributable to low temperature, which is an indirect marker of patient survival. Over the past 30 years, there has been a gradual shift in deaths toward older age groups (80+) globally, and this trend has particularly occurred in East Asia and the Pacific and Latin America and Caribbean regions. For the European, Central Asian and North American regions, the death rate showed positive U-shaped or inverted U-shaped changes with a slight increase. In 2019, more than half of the deaths occurred in the older age group in these two regions, which is far greater than in other regions. In South Asia and Saharan Africa, deaths attributed to low temperature are still concentrated in people under 80 years of age (>75%), and this trend has not improved significantly in the past 30 years. A similar trend was observed for the distribution of DALYs (Figure 3B). The male population dies at a younger age than does the female population for CVD across all regions. Supplementary Figure S3 shows that men have higher CVD age-standard mortality and DALY rates attributed to low temperature than women in all regions. A more favorable decrease in mortality was found in females than in males in all of the regions except North America. Therefore, health concerns for the male population should be raised and strengthened.

### Effects of age, period, and cohort factors on death and DALY loss

**Figure 4:**
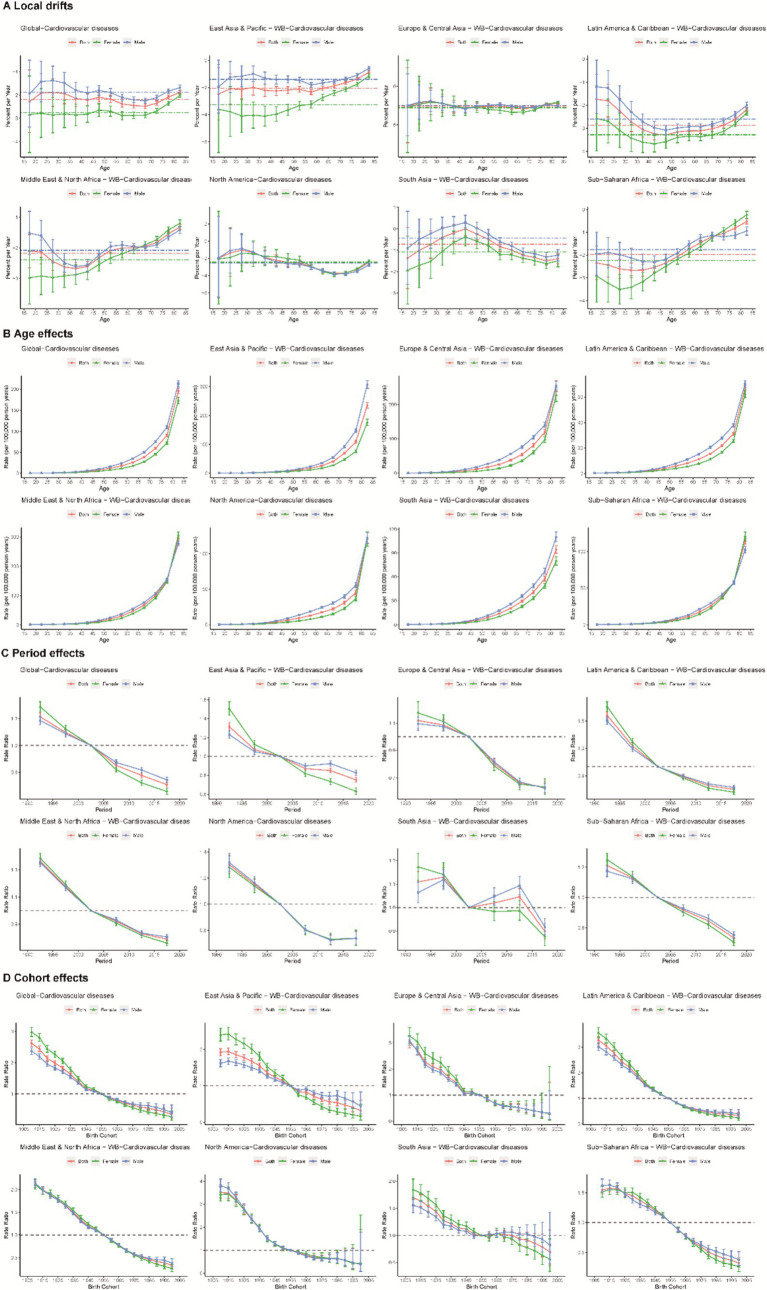
Local drifts **(A)**, age **(B)**, period **(C)**, and cohort effects **(D)** on CVD mortality attributed to low temperature across different regions.

Figure 4 shows the local drift in mortality for each age group in different regions and different sex categories, highlighting significant differences across the globe and in the seven regions. A greater decrease was detected in females than in males. A flat trend in the mortality decline rate for all age groups was observed in Europe, Central Asia and North America, with similar trends between different sex categories. An increasing trend of local drifts across different ages was found in the East Asia and Pacific and Sub-Saharan Africa regions, and a “U-shaped” trend was observed in the Latin America and Caribbean and Middle East and North American regions. However, an inverted U-shaped trend in the local drift of CVD mortality attributed to low temperature was found in South Asia, with the middle-aged group (35–50 years old) exhibiting the slowest or even stagnant trend.

A similar pattern of age effects was found across regions, with the risk of CVD death increasing with age. The disease burden was more severe in East Asia, the Pacific, and the MENA region for those aged 70 years or older than in other regions. The period effect generally showed a declining risk of CVD mortality across different regions, except for South Asia. Females presented a higher CVD mortality risk attributed to low temperature in the period from 1990 to 2004, and a lower risk was found thereafter. Globally, there was an overall declining risk in successively younger birth cohorts. Similar to period effects, declining cohort effects were more noticeable in North America. The mortality in North America progressively improved in those born after the 1900s, whereas the risk in the Sub-Saharan Africa region did not significantly decrease until after the 1925 cohort.

In addition, the effects of age, period, and cohort factors on trends in DALY and local drift changes were essentially the same as the trends in mortality, as detailed in Supplementary Figure S4.

### Age-period-cohort effect for representative countries

**Figure 5:**
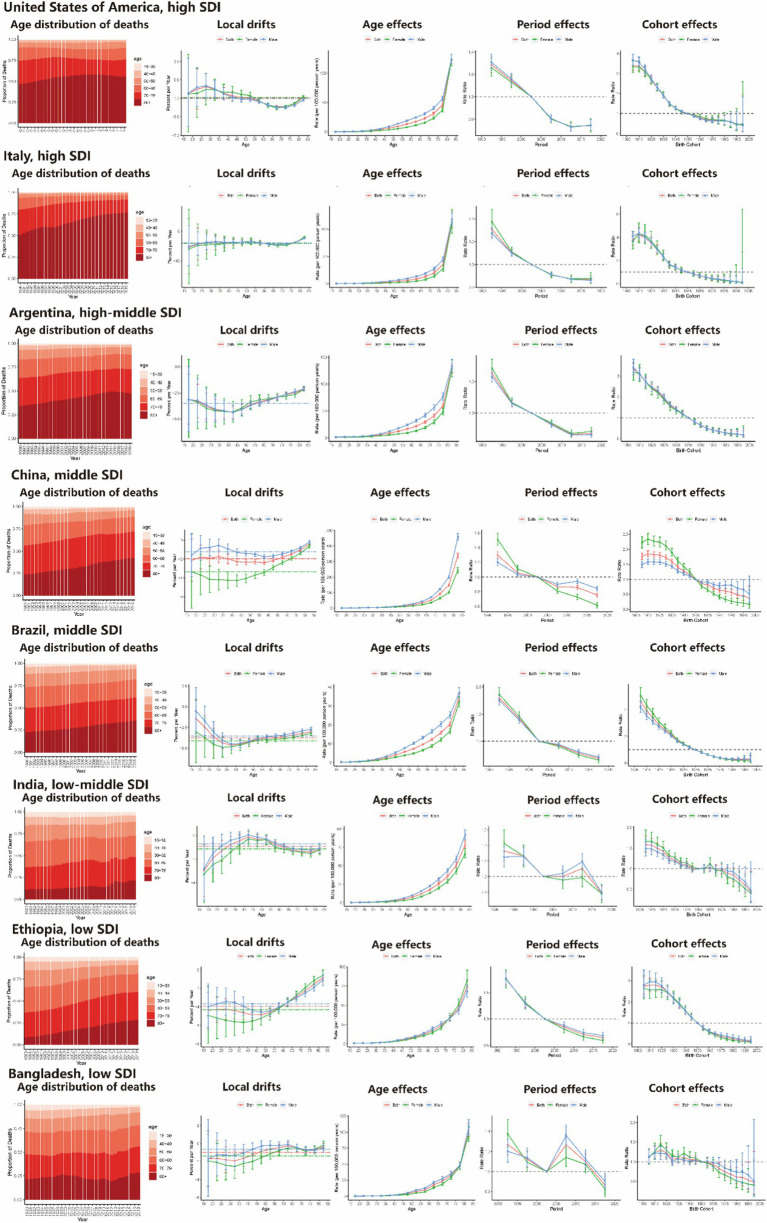
Age distribution of death and age-period-cohort effects attributed to low temperature on exemplar countries across SDI quintiles. SDI, sociodemographic index.

Several representative countries were selected from the 204 countries and regions with different ASR risks (2019) to better depict the main trends in CVD mortality and DALY loss globally through age-period-cohort effects. Figure 5 illustrates the age distribution of the population, the local drift and age-period-cohort effects of mortality for CVD and its subtypes attributable to low temperature in eight representative countries (high-SDI countries, such as the United States and Italy; high-middle-SDI countries, such as Argentina; middle-SDI countries, such as China and Brazil; low-middle-SDI countries, such as India; and low-SDI countries, such as Ethiopia and Bangladesh). The main trends of DALYs in these countries are shown in Supplementary Figure S5.

The United States is a typical North American and high-SDI country with a shift in CVD mortality toward the older (80+) age group. This trend was particularly pronounced in the female cohort. The risk of death rapidly increased after the age of 70. However, there was an overall favorable period in the birth cohort. Italy is a typical high-SDI country in the European region with age-period-cohort effects similar to those in the United States, and a much larger proportion of deaths occurred in older age groups. In the context of the energy crisis, the health status of the population in European countries in winter should receive more attention. Argentina, China, and Brazil are high-middle-SDI and middle-SDI countries. As shown in Figure 5, similar age distributions of death and APC effects were observed in these three countries. The death rate in Argentina was greater in the older population than that in China and Brazil, and the distribution of deaths across age and APC effect was close to that in high-SDI countries. China accounts for more than 35% of the CVD deaths attributed to low temperature globally, and there has been a relatively rapid shift in the distribution of CVD-related deaths toward older age groups in the last 30 years. However, deaths in China are still more concentrated among people under 80 years of age, as in other middle- and low-SDI countries, unlike in high-SDI countries. This may imply a rigorous disease burden profile in the future. India, Ethiopia and Bangladesh were examples of typical low-middle- and low-SDI countries. In Ethiopia, which is the most populous low-SDI country in East Africa, the trend of the age distribution of deaths and APC effects was similar to that in China, with a relatively rapid shift in the distribution of deaths toward older age groups. However, in India, a low-middle-SDI country, the APC effects and local drifts were more similar to those in Bangladesh (a low-SDI country), which has a similar geography and climate, and presented a relatively slow shift in the distribution of deaths toward older age groups and a gradual decline in period and cohort risk. The main trends in CVD mortality and DALY loss through age-period-cohort effects for all countries showed similar trends and are presented in Supplementary Figure S5.

### Mortality levels in countries and regions with different SDI levels and geographic locations

**Figure 6:**
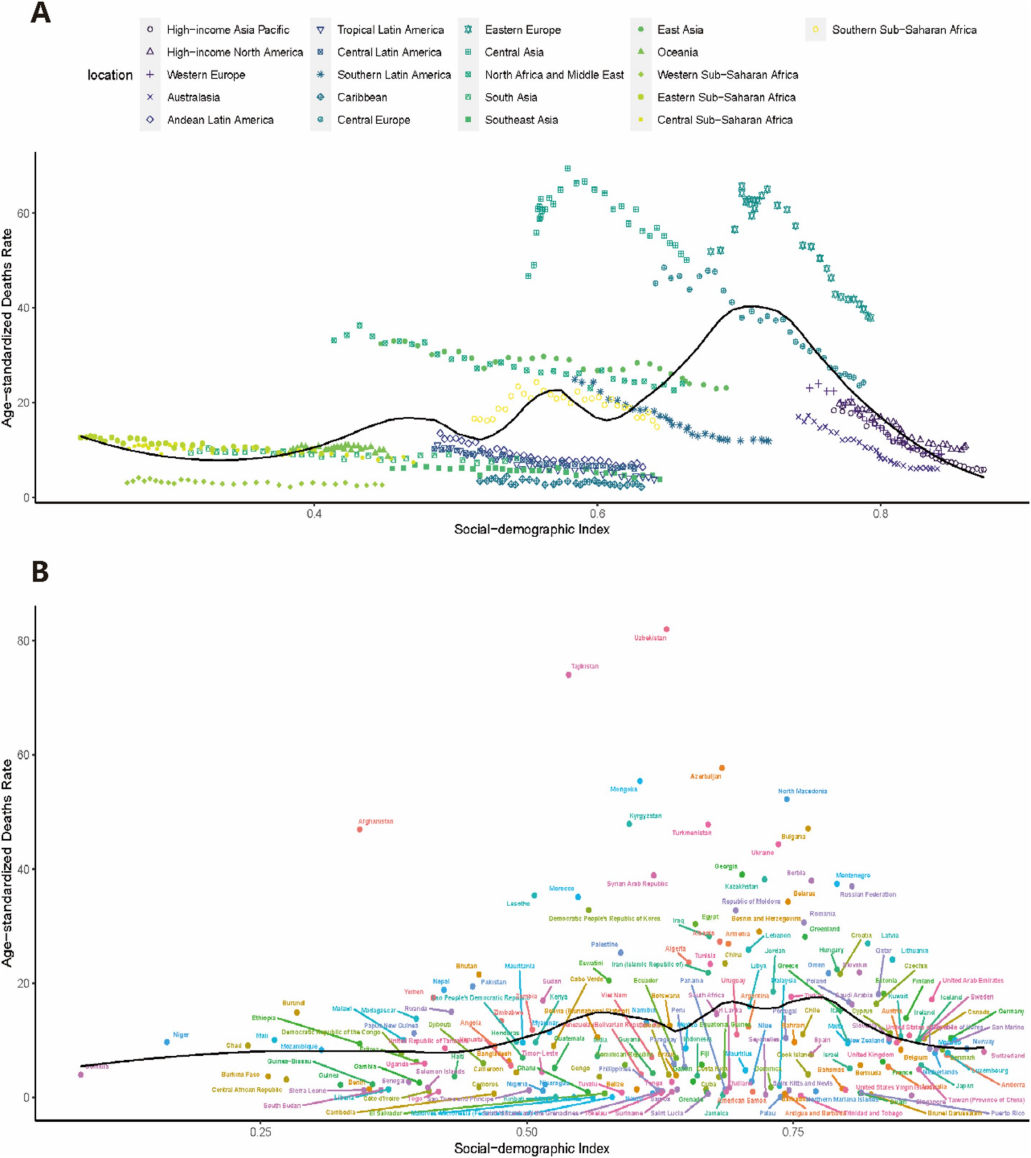
Trends in age-standardized CVD mortality in 12 GBD regions by sociodemographic index **(A)** and age-standardized CVD mortality in 204 countries in 2019 **(B)**. Each colored dot represents a country, with a solid black line depicting the non-linear fitting trend. Countries or territories were classified according to the locations **(A)**. CVD, cardiovascular disease; GBD, global burden of disease.

Figure 6 and Supplementary Figure S6 show the distributions of mortality and DALYs in 21 GBD regions and 204 countries with different SDI levels for CVD and its subtypes, respectively. Eastern Europe, Central Europe, and Central Asian countries with higher SDI levels had higher CVD mortality rates, and countries in Saharan Africa with lower SDI levels had lower CVD mortality rates attributed to low temperature. Those countries in East Asia, North Africa and the Middle East had both moderate SDI levels and CVD mortality attributed to low temperature. The distribution of mortality among the 204 countries suggests that SDI levels have a limited effect on the risk of CVD mortality attributable to low temperature. Geographic location and climatic environment may have a greater effect on mortality. Similar trends were seen in the distribution of DALYs.

## Discussion

Although low temperature is not one of the major risk factors for CVD mortality and DALYs, it is one of the most worldwide influential factors (Supplementary Figure S7). Despite the great variations in the trend and magnitude of CVD burden attributable to low temperature between different regions and countries (20), steps have been taken for the prevention and control of CVDs in the last 30 years. However, CVDs undoubtedly remain a significant challenge for human health in the future. The intensification of the energy crisis (8) has further worsened the winter heating situation in some countries and regions, causing a greater health burden. Therefore, increased attention should be given to the burden of CVDs caused by low temperature.

Consistent with previous GBD publications and similar studies, the present study suggested that older populations are experiencing an increasing burden of CVDs (9). Despite achievements in disease prevention and control over the past three decades, health disparities in CVDs attributable to cold temperature appear to be widening across the globe and will likely increase the global burden of CVDs. Analysis of GBD mortality and DALY data suggested that countries and regions with higher SDIs have higher disease mortality but also faster rates of mortality decline. However, geographic location and climatic environment may play a more significant role in shaping mortality than economic development. In addition, men are at greater risk of death than women are and die at a younger age. Therefore, disease burden estimates need to consider all aspects—development level, natural environment, age and sex—to develop individualized prevention and control strategies for CVDs.

For the first time, we used an APC model to analyze temporal trends in mortality and DALYs for CVD and its subtypes attributable to low temperature globally and in 204 countries. Compared with previous GBD publications (9, 22), this study provides a more refined understanding of CVD trends to generate insightful public health perspectives. Temporal and cohort effects differentiate the sources of mortality trends based on time periods and birth cohorts in each country, thus informing the effectiveness of relevant health care services (21, 23). Another important advancement, the estimation of local drift values, allows capturing temporal trends in mortality for each age group and adjusting for period effects (22, 24). Nevertheless, the use of overall rates to assess changes in mortality and DALYs can ignore important information about differences between age groups, time periods, and birth cohorts. Therefore, APC models were used to facilitate a deeper understanding of CVD incidence trends. The combination of various factors, such as geographical location, SDI level, disease subtype, and sex, facilitates the exploration of the causes of mortality and DALY variations and suggests targeted prevention and treatment strategies.

A distinctive feature of the burden analysis of CVD and its subtypes attributable to low temperature is the high sensitivity to geographical location and climatic environment. Therefore, natural environmental factors need to be considered in addition to the level of economic and social development. Most of the eight countries within the South Asia region have low and moderate-low SDI levels. Consequently, the mortality decline rate is slow in these countries (net drift = −0.72 [−0.89, 0.55]). However, CVD mortality attributable to low temperature in these countries is also much lower than the world average due to their location in tropical and subtropical climate regions with high annual temperatures (4, 25). The same phenomenon has also been observed in Saharan Africa. In contrast, countries such as Sweden, Finland, and Canada have relatively high mortality rates due to higher dimensionality and colder winter climates (26). However, higher levels of SDI in these countries have led to a rapid decline in mortality over the last 30 years. Another important finding is that the burden of CVD and its subtypes attributable to cold temperature is greater in men than in women in countries and regions with different SDI levels and natural environments. This finding is consistent with the results of previous studies (27, 28). However, the present study further disaggregates the various scenarios to make these findings more applicable. Therefore, policy-makers should be more concerned about the health risks associated with low temperature for the male population. In addition, there was an unfavorable trend in the period effect of hypertensive heart disease due to cold temperatures in North America. Furthermore, birth cohort effects have shown little improvement over the past 30 years. However, European regions with similar natural environments and levels of socioeconomic development showed favorable trends. This suggests that medical resources for CVDs are underinvested in some countries.

This study provides an in-depth analysis of CVD trends using GBD data. The APC model can also be applied to other NCDs to analyze disease trends by age, period, and population to obtain a clearer picture of the effectiveness of health system responses. This approach is superior to traditional epidemiological indicators for tracking progress toward sustainable development goals (SDGs) in certain countries.

This study has several limitations. First, our analysis suffers from the limitations derived from the GBD Study due to the limited availability of raw data from low- and middle-income countries (LMICs) and estimates from high-resource settings. Comprehensive data on disease mortality and DALY loss in many LMICs are urgently needed. Second, GBD studies have primarily focused on mutually exclusive and individual causes of death. For middle-aged and older adults with multiple risk factors, CVD deaths and DALY loss may not be attributable to low temperature. Therefore, the actual mortality in GBD studies may be underestimated. The real situation may be more severe. Third, this study analyzed mortality at the national level and did not capture local differences. A more refined analysis using local data could identify trends that vary across regions within countries due to their varying levels of development. Finally, due to the unavailable data on latitude, average annual temperature, or annual minimum temperature, the potential relationship between these factors and low temperature on CVD mortality were not analyzed in this article.

## Conclusion

Mortality and DALY loss from CVDs attributable to low temperature showed an overall decreasing trend globally, with favorable trends in the period and birth cohorts. Countries or regions with higher SDIs tend to have greater disease mortality but faster rates of mortality decline. However, the disease burden profile is influenced by both the level of economic and social development and the natural environment. Therefore, individualized assessments and prevention strategies are needed based on specific country conditions. Men have a greater disease burden globally and die at a younger age than women. These findings suggest that health disparities between sexes and regions are widening, leading to a heavy disease burden. Health authorities and policy-makers need to consider ways to better allocate resources, increase access to health care resources, and control variable risk factors in a timely manner.

## Data Availability

Publicly available datasets were analyzed in this study. This data can be found at: http://ghdx.healthdata.org/gbd-2019.

## Footnotes

^1^http://analysistools.nci.nih.gov/apc/
