# Supplementary material for: Global, regional, and national temporal trends in mortality and disability-adjusted life years for cardiovascular disease attributable to low temperature during 1990–2019: an age-period-cohort analysis of the global burden of disease 2019 study
Source: Front Public Health. 2024 Oct 10;12:1414979. doi: 10.3389/fpubh.2024.1414979 (PMC11500463; doi:10.3389/fpubh.2024.1414979)
Supplement: Supplementary file 1 [file Data_Sheet_1.docx]

**Global, regional, and national temporal trends in mortality and disability-adjusted life years for cardiovascular disease attributable to low temperature during 1990–2019: an age-period-cohort analysis of the global burden of disease 2019 study**


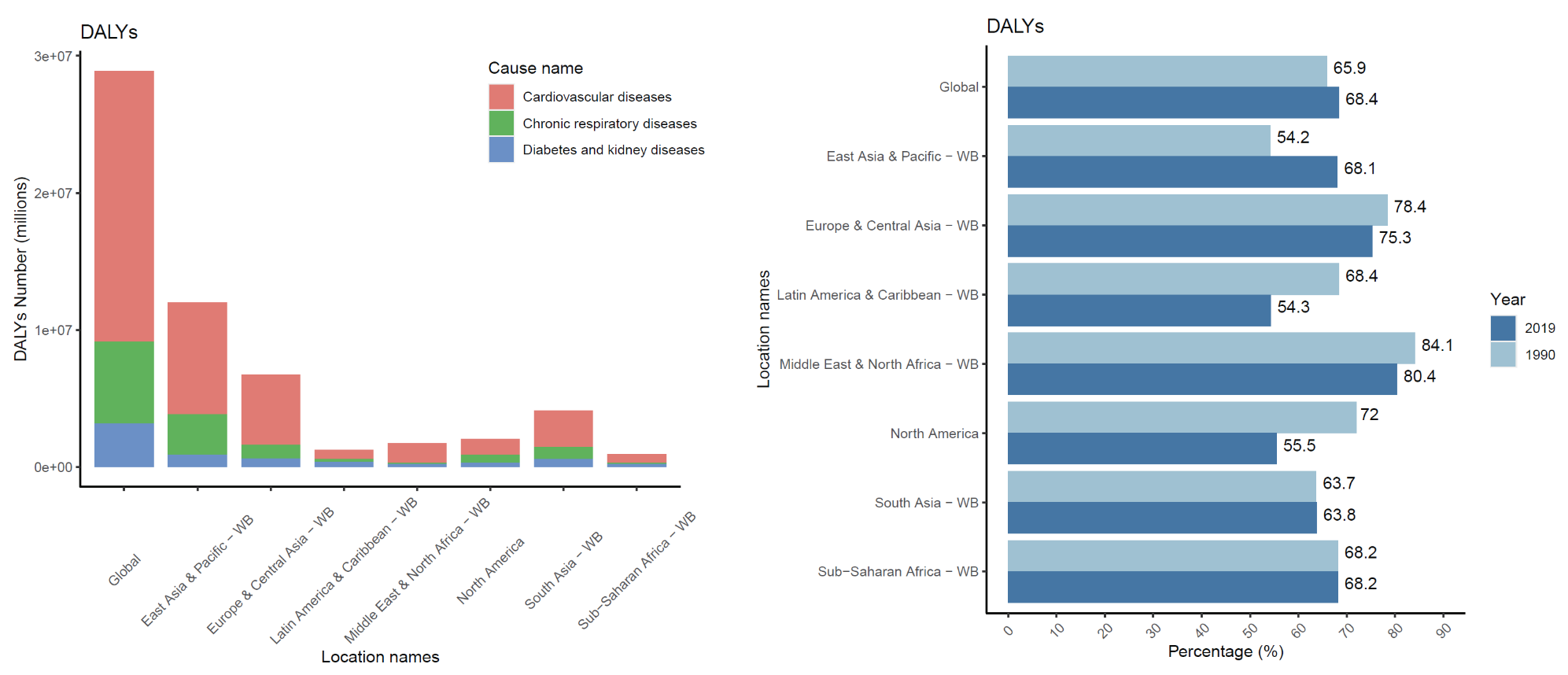


**Figure S1.** CVD is the leading cause of global DALYs attribute to low temperature from NCDs in the population worldwide. (A). The number of DALYs in 2019 for NCDs due to low temperature, and cardiovascular diseases account for the largest population. (B), Change in the proportion of CVD DALYs across different regions, 1990-2019. CVD, Cardiovascular disease; NCD, non-communicable disease; DALY, disability-adjusted life year.


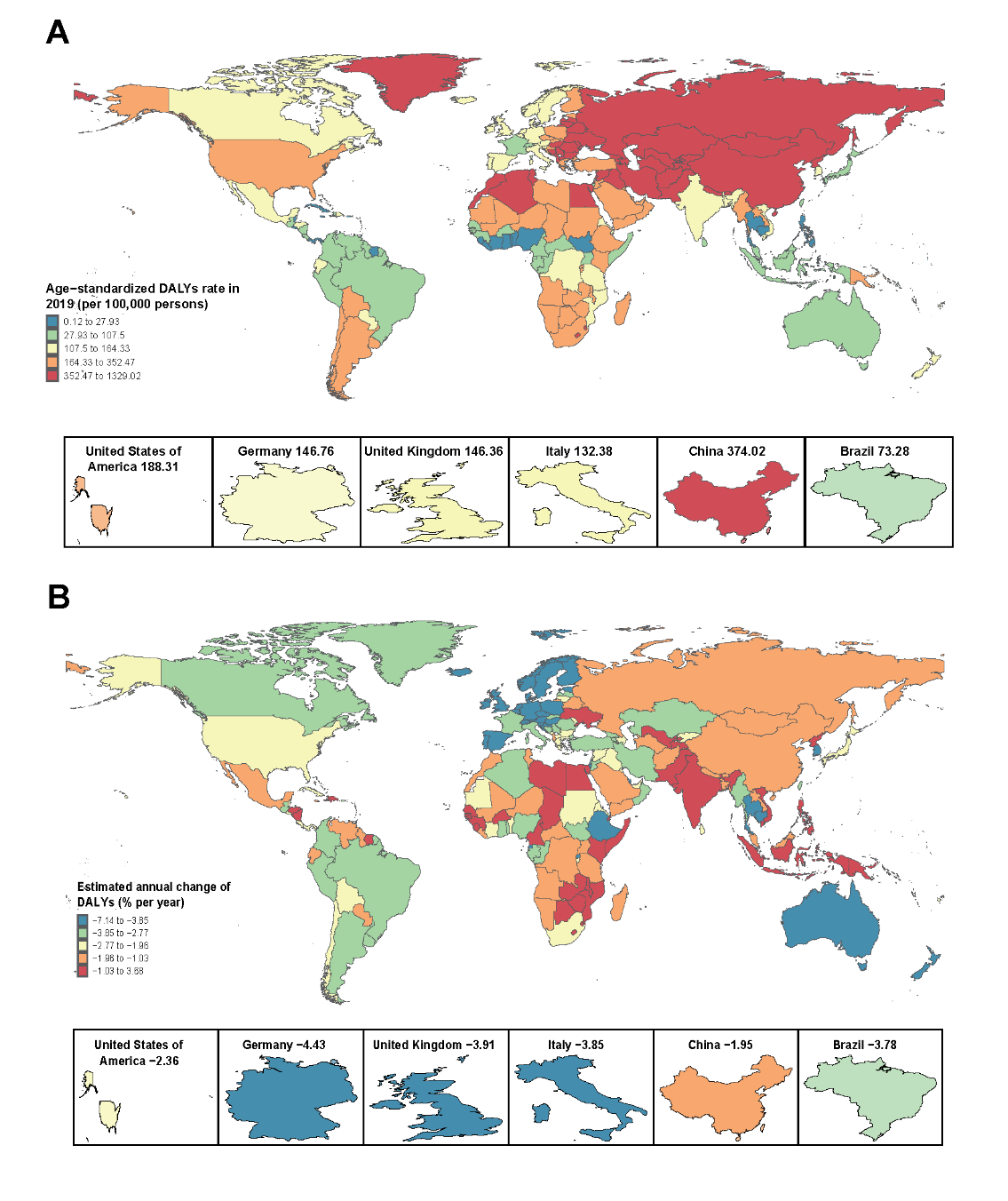


**Figure S2. The all-age DALYs in 2019 (A) and net drift of DALYs during 1990-2019 (B) for CVDs in 204 countries and territories due to low temperature.** (A) World map of all-age DAYLs for CVDs. In 2019, the global all-age DALYs was 255.5 (95%UI 207.3-309.22) was per 100,000 population. (B). World map of the net drifts for DALYs of CVDs, i.e., estimated annual percentage change of DALYs from age-period-cohort model. Net drift captures components of the trends attributable to calendar time and successive birth cohorts. The global net drift of CVDs DALYs was -2.17[95%CI -2.26 to -2.09] . CVD, cardiovascular disease.


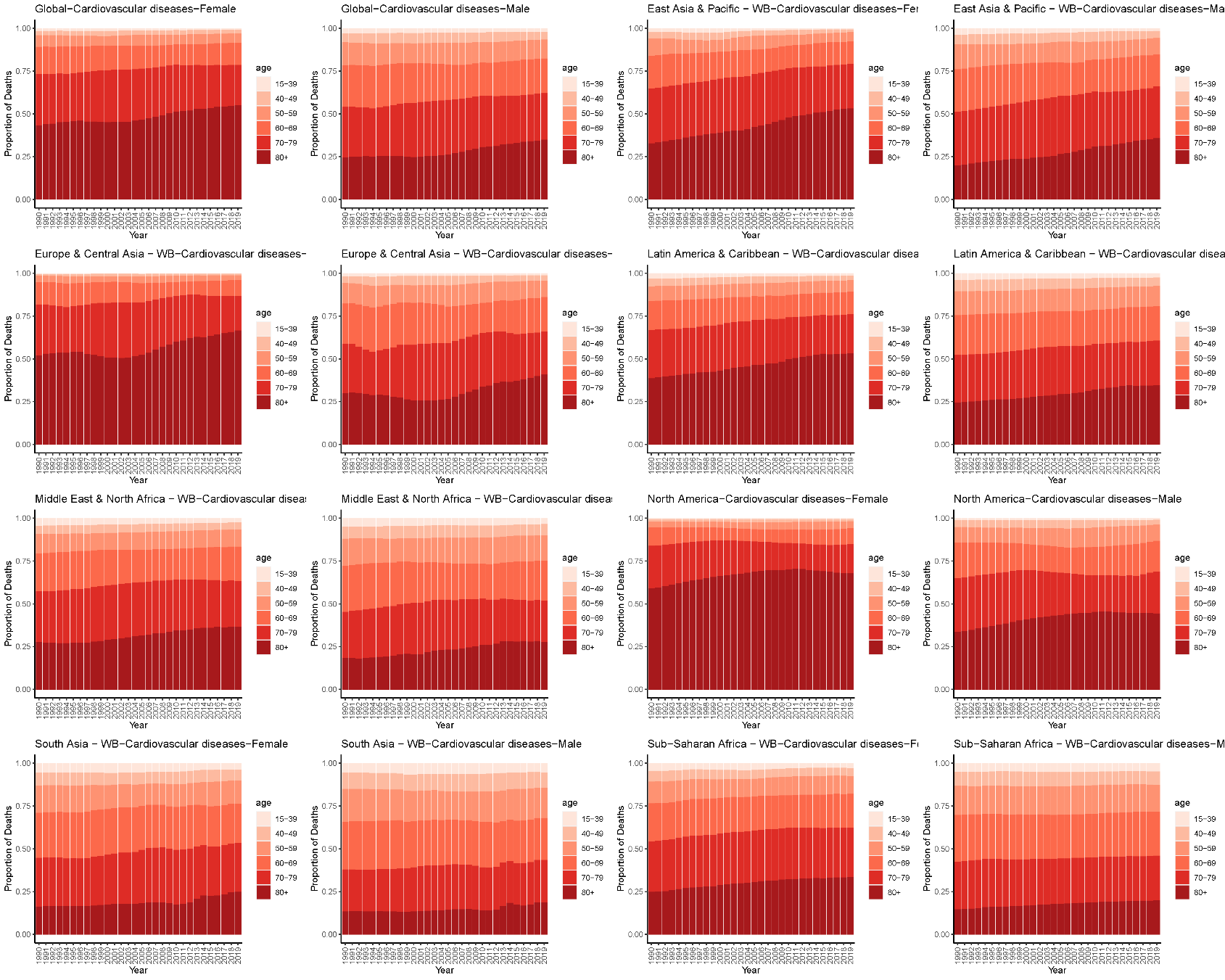


**Figure S3A. Age and sex distribution of CVD death due to low temperature.** CVD, cardiovascular disease.


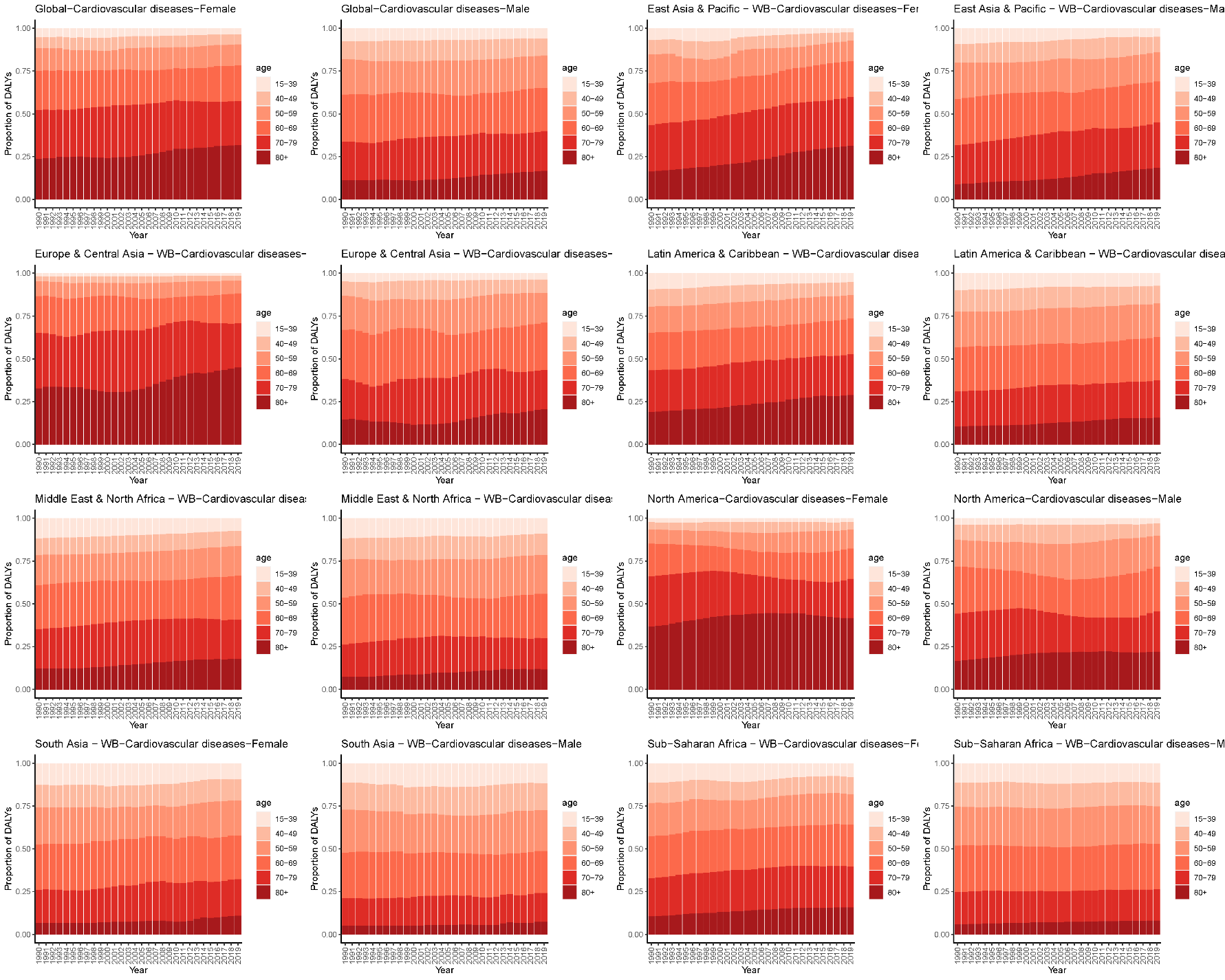


**Figure S3B. Age and sex distribution of CVD DALYs due to low temperature.** CVD, cardiovascular disease. DALY, disability-adjusted life years.


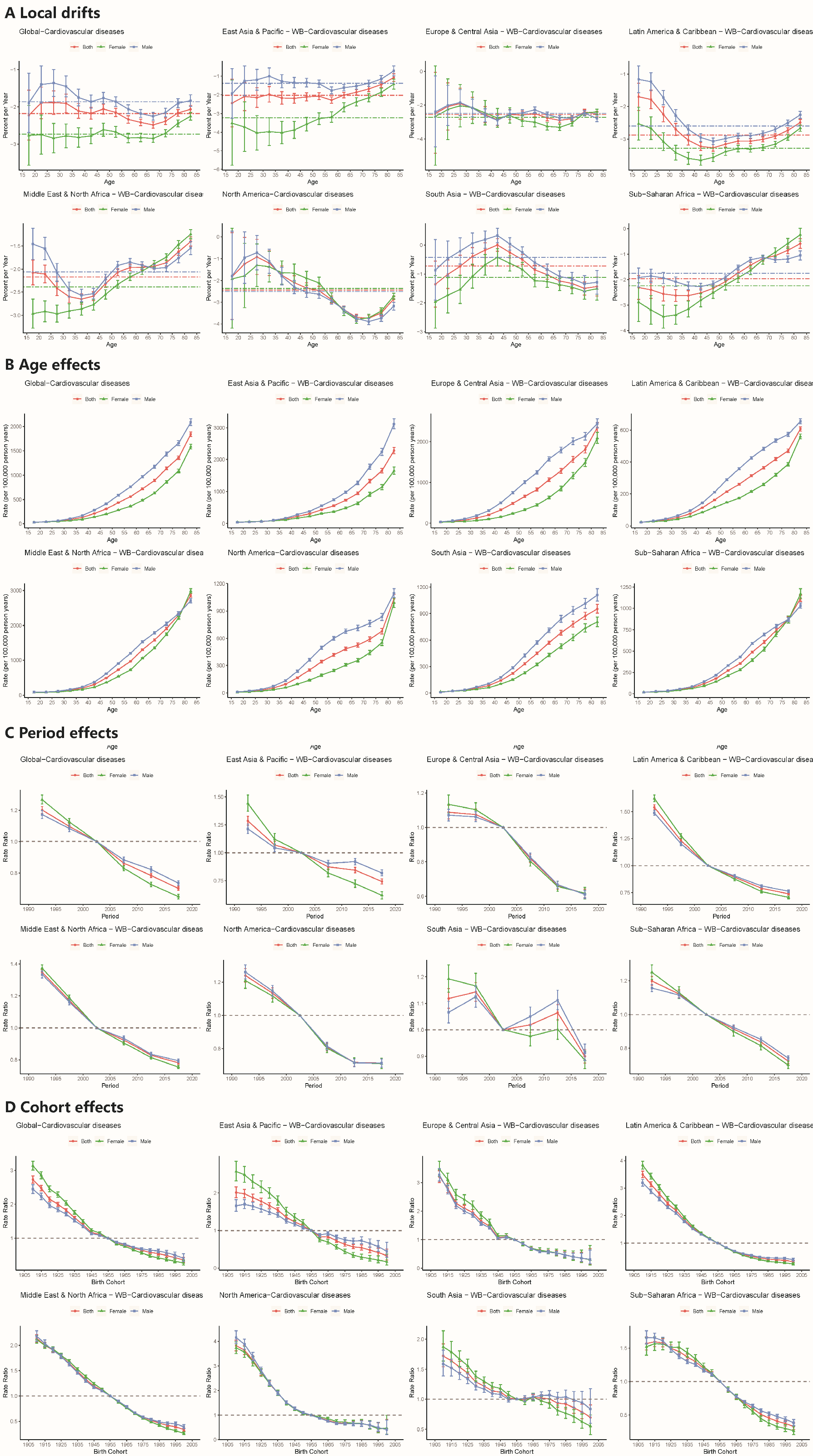

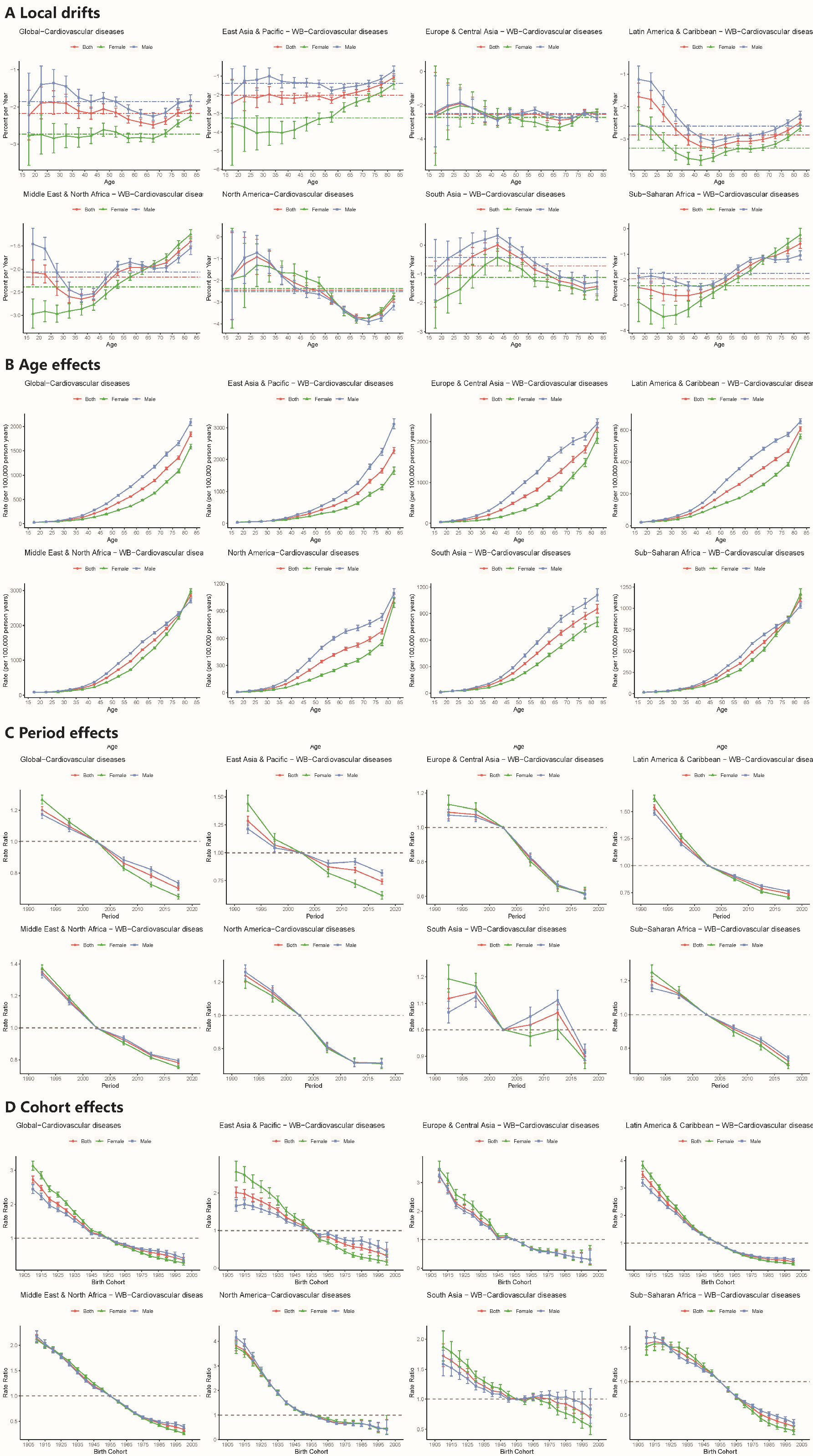


**Figure S4** **Local drifts, age, period, and cohort effects on DALYs of CVDs attributed to low temperature across different regions.**

**
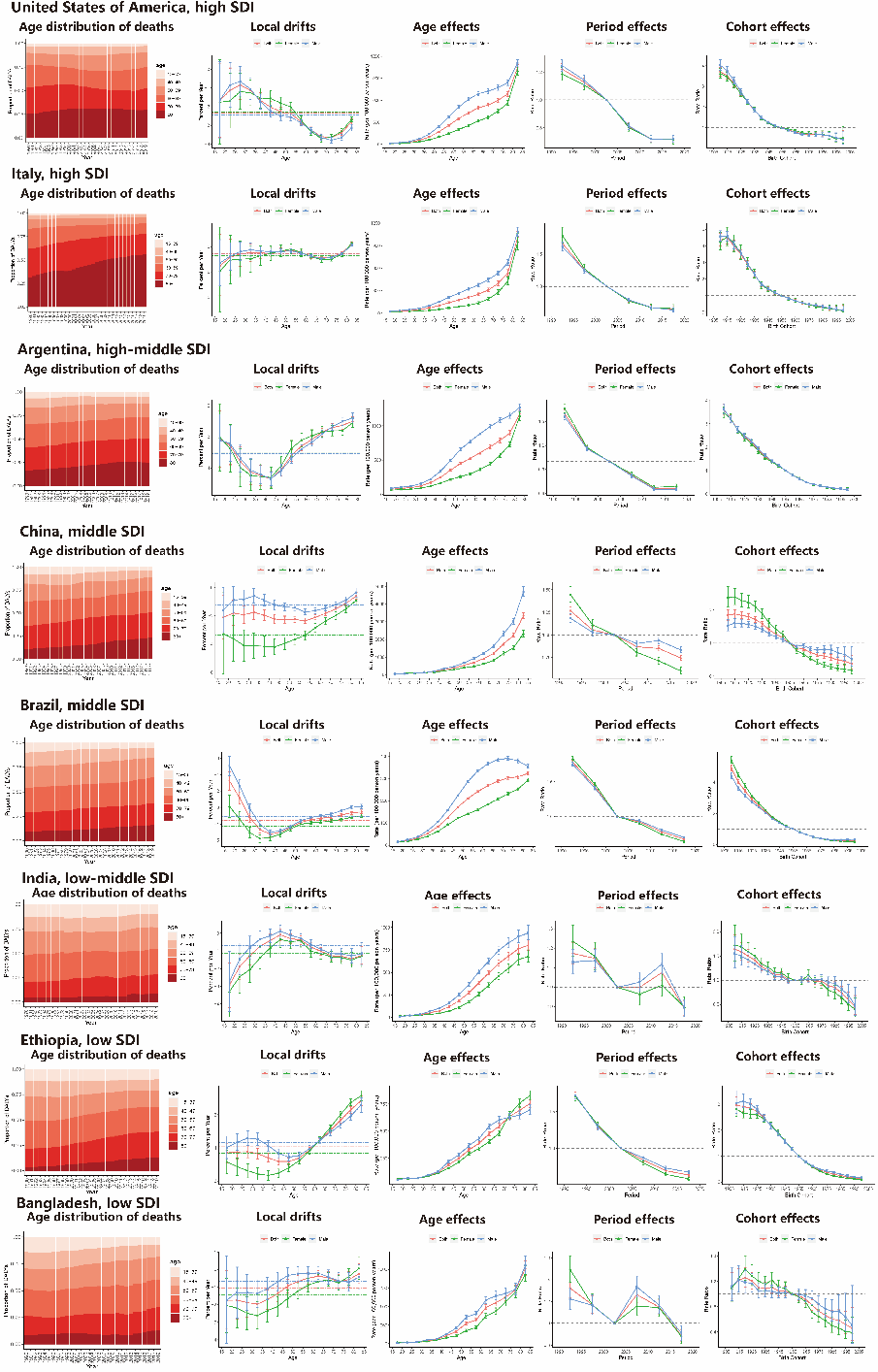
**

Figure S5 **Age distribution of DALYs and age-period-cohort effects attributed to low temperature on exemplar countries across SDI quintiles.** SDI, socio-demographic index.


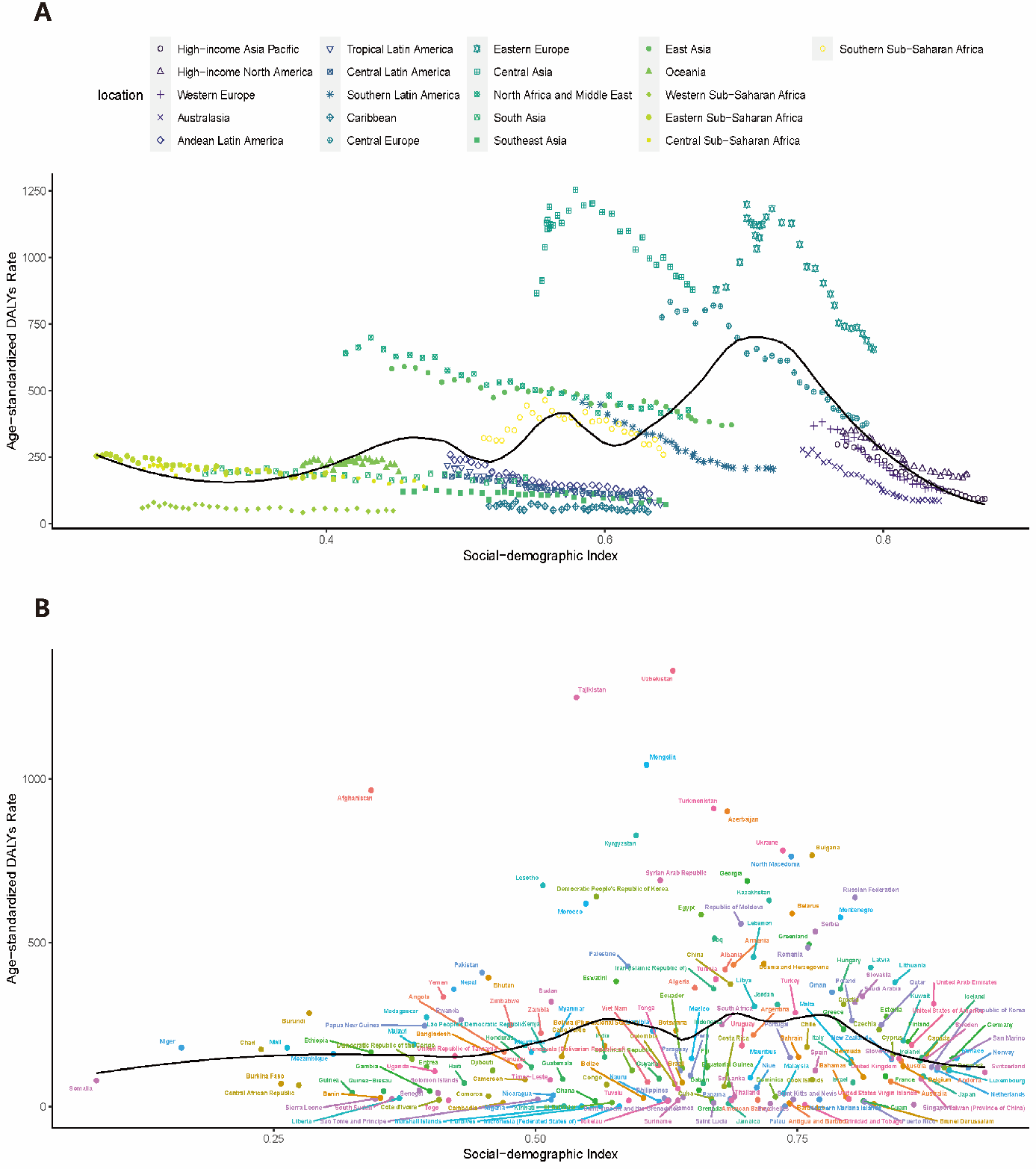


**Figure S6. Trends in age-standardized CVD DALYs attributed to low temperature in 12 GBD regions by socio-demographic index (A) and age-standardized CVD mortality in 204 countries in the year of 2019 (B).** For each region (A), points from left to right depict estimates from each year from 1990 to 2019.


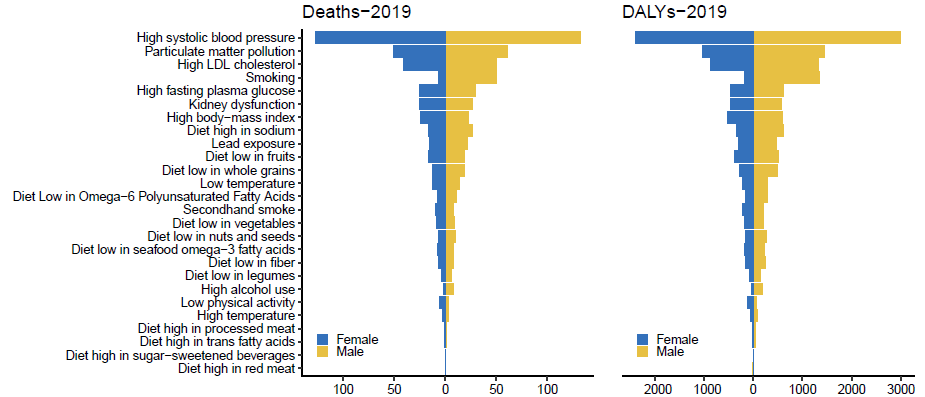


**Figure S7 GBD-listed risk factors for cardiovascular disease (mortalities and DALYs).** LDL, low-density lipoprotein.

# Table S1 Trends in CVDs DALYs attributed to low temperature across different regions, 1990−2019.

|  | **Global** | | **East Asia & Pacific** | | **Europe & Central Asia** | | **Latin America & Caribbean** | | **Middle East & North Africa** | | **North America** | | **South Asia** | | **Sub-Saharan Africa** | |
| --- | --- | --- | --- | --- | --- | --- | --- | --- | --- | --- | --- | --- | --- | --- | --- | --- |
|  | **1990** | **2019** | **1990** | **2019** | **1990** | **2019** | **1990** | **2019** | **1990** | **2019** | **1990** | **2019** | **1990** | **2019** | **1990** | **2019** |
| **DALYs** |  |  |  |  |  |  |  |  |  |  |  |  |  |  |  |  |
| Number, n× 1,000 | 16273.58(13155.47,19500.69) | 19768.99(16039.59,23925.95) | 5814.74(4760.94,6979.77) | 8171.41(6556.35,10049.78) | 6141.81(4407.87,8064.26) | 5089.29(3707.19,6725.28) | 640.45(506.62,782.42) | 686.56(534.6,848.3) | 807.09(580.56,1022.94) | 1384.73(988.48,1792.82) | 1205.9(938.85,1495.82) | 1132.3(904.55,1362.26) | 1204.43(591.56,1794.14) | 2630.71(1419.75,3835.3) | 438.47(308.11,573.68) | 646.68(444.42,869.54) |
| Percentage of global, % | 100 | 100 | 35.73 | 41.33 | 37.74 | 25.74 | 3.94 | 3.47 | 4.96 | 7 | 7.41 | 5.73 | 7.4 | 13.31 | 2.69 | 3.27 |
| Percent change of deaths 1990–2019, % | 21.48(10.65,33.52) |  | 40.53(18.63,66.15) |  | -17.14(-22.87,-10.75) |  | 7.2(-1.95,17.84) |  | 71.57(47.38,97.74) |  | -6.1(-10.82,-1.36) |  | 118.42(75.9,218.17) |  | 47.49(29.2,69.58) |  |
| **All-age DALY rate** | | | | | | | | | | | | | | | | |
| Rate per 100,000 | 304.19(245.9,364.51) | 255.5(207.3,309.22) | 310.17(253.96,372.31) | 347.17(278.55,426.97) | 719.74(516.55,945.03) | 553.32(403.05,731.18) | 145.97(115.47,178.33) | 105.71(82.31,130.62) | 311.35(223.96,394.62) | 301.95(215.54,390.93) | 429.27(334.2,532.47) | 310.59(248.12,373.67) | 106.92(52.51,159.27) | 140.99(76.09,205.55) | 85.72(60.24,112.16) | 57.8(39.72,77.72) |
| Percent change of rate 1990–2019, % | -16.01(-23.49,-7.68) |  | 11.93(-5.51,32.34) |  | -23.12(-28.44,-17.2) |  | -27.58(-33.76,-20.39) |  | -3.02(-16.69,11.77) |  | -27.65(-31.28,-23.99) |  | 31.87(6.2,92.09) |  | -32.57(-40.93,-22.48) |  |
| **Age-standardized DALY rate** | | | | | | | | | | | | | | | | |
| Rate per 100,000 | 426.07(341.84,510.95) | 245.53(199.53,297.25) | 447.12(368.93,534.44) | 269.9(217.14,330.93) | 583.42(418.67,765.48) | 322.1(233.91,428.11) | 238.76(189.11,291.3) | 103.59(80.62,127.97) | 622.92(452.77,795.36) | 436.55(314.51,558.69) | 345.23(268.67,428.09) | 185.11(148.51,222.38) | 200.42(97.65,298.16) | 177.17(96.02,257.36) | 190(135.45,246.65) | 136.24(94.34,180.44) |
| Percent change of rate 1990–2019, % | -42.37(-47.42,-36.69) |  | -39.64(-48.87,-28.69) |  | -44.79(-48.9,-40.32) |  | -56.61(-60.34,-52.45) |  | -29.92(-38.69,-20.44) |  | -46.38(-49.08,-43.67) |  | -11.6(-28.37,28.07) |  | -28.29(-35.85,-18.81) |  |
| **APC model estimates** | | | | | | | | | | | | | | | | |
| Net drift of mortality y, % per year | -2.17(-2.26,-2.09) |  | -2.03(-2.17,-1.89) |  | -2.54(-2.76,-2.32) |  | -2.87(-2.93,-2.81) |  | -2.17(-2.22,-2.13) |  | -2.45(-2.64,-2.26) |  | -0.73(-0.84,-0.61) |  | -1.96(-2.04,-1.88) |  |

# Table S2. Number of death, all-age mortality, age-standardized mortality and net drift of mortality distribution attributed to low temperature in 204 countries and territories.

| **Regions** | **Countries** | **Number of deaths in 2019** | **All-age mortality rate, per 100,000 in 2019** | **Age-standardized mortality rate** | **Net drift of mortality**  **y, % per year** |
| --- | --- | --- | --- | --- | --- |
| East Asia | Cook Islands | 2(0,2) | 5.29(0.65,12.56) | 4.03(0.49,9.6) | -1.91(-24.24,27) |
| East Asia | Democratic People's Republic of Korea | 9620(6608,13476) | 36.67(25.19,51.37) | 32.81(22.61,45.91) | -0.07(-0.33,0.18) |
| East Asia | Lao People's Democratic Republic | 346 (167,588) | 4.84(2.33,8.21) | 9.81(4.85,16.39) | -1.6(-2.5,-0.69) |
| East Asia | Micronesia (Federated States of) | 0(0,0) | 0.01(0,0.09) | 0.02(0,0.15) | -2.37(-17.31,15.27) |
| East Asia | Niue | 0(0,0) | 3.53(0.26,10.22) | 2.79(0.2,8.11) | -2.67(-61.93,148.83) |
| East Asia | Republic of Korea | 6947 (4779,9429) | 13.01(8.95,17.66) | 8.54(5.87,11.52) | -7.1(-7.62,-6.57) |
| East Asia | Taiwan (Province of China) | 1973 (1378,2694) | 8.35(5.84,11.4) | 4.89(3.41,6.68) | -3.21(-3.93,-2.48) |
| East Asia | Tokelau | 0 (0,0) | 0.7(0,1.48) | 0.8(0,1.72) | -1.26(-88.87,775.67) |
| East Asia | Viet Nam | 5586 (2584,9625) | 5.8(2.68,9.99) | 7.08(3.32,12.18) | -1.02(-1.36,-0.69) |
| East Asia | Brunei Darussalam | 1(0,3) | 0.21(0.02,0.61) | 0.48(0.04,1.39) | -4.65(-22.31,17.04) |
| East Asia | China | 392404(314400,484446) | 27.59(22.1,34.06) | 23.47(18.86,28.75) | -1.96(-2.17,-1.75) |
| East Asia | Mongolia | 962 (470,1620) | 28.41(13.86,47.82) | 55.44(26.75,91.89) | -1.23(-1.87,-0.58) |
| East Asia | Guam | 1 (0,3) | 0.84(0,1.75) | 0.77(0,1.6) | -3.39(-19.52,15.98) |
| East Asia | Nauru | 0 (0,0) | 0.23(0,0.63) | 0.66(0,1.87) | -2.17(-46.45,78.72) |
| East Asia | Northern Mariana Islands | 0(0,1) | 1.06(0.18,1.92) | 1.06(0.17,1.92) | -1.57(-26.39,31.61) |
| East Asia | Palau | 0(0,0) | 0.03(-0.11,0.23) | 0.03(-0.1,0.23) | -2.67(-40.78,59.95) |
| East Asia | Timor-Leste | 29 (9,59) | 2.2(0.68,4.39) | 4.46(1.41,8.76) | 1(-3.29,5.48) |
| East Asia | Singapore | 25(0,48) | 0.44(0,0.85) | 0.34(0,0.66) | -5.03(-12.85,3.49) |
| East Asia | Fiji | 36(12,67) | 3.91(1.33,7.4) | 5.74(1.99,10.83) | -2.18(-4.88,0.6) |
| East Asia | Cambodia | 60(22,107) | 0.36(0.13,0.65) | 0.62(0.23,1.12) | -4.2(-5.79,-2.58) |
| East Asia | Myanmar | 4475(2891,6398) | 8.18(5.29,11.7) | 11.44(7.44,16.22) | -3.08(-3.32,-2.83) |
| East Asia | Thailand | 945 (412,1735) | 1.35(0.59,2.47) | 0.96(0.42,1.77) | -3.86(-4.39,-3.32) |
| East Asia | Indonesia | 6482(2869,11483) | 2.5(1.11,4.43) | 3.79(1.65,6.73) | -0.67(-0.9,-0.45) |
| East Asia | Marshall Islands | 0(0,0) | 0.01(0,0.06) | 0.02(0,0.13) | -1.36(-22.8,26.04) |
| East Asia | Solomon Islands | 5(1,12) | 0.74(0.15,1.9) | 1.72(0.34,4.37) | -2.19(-6.4,2.21) |
| East Asia | Japan | 27348 (20190,33227) | 21.4(15.8,26) | 5.64(4.37,6.75) | -2.69(-3.45,-1.93) |
| East Asia | Tuvalu | 0 (0,0) | 0.64(0,1.38) | 0.84(0,1.79) | -1.59(-49.21,90.68) |
| East Asia | New Zealand | 862(611,1117) | 19.17(13.58,24.86) | 9.88(7.05,12.84) | -4.63(-6.26,-2.98) |
| East Asia | American Samoa | 0 (0,1) | 0.75(0,1.48) | 0.98(0,1.95) | -0.91(-26.09,32.85) |
| East Asia | Philippines | 829(363,1411) | 0.74(0.32,1.26) | 1.24(0.56,2.07) | 3.54(2.75,4.34) |
| East Asia | Kiribati | 0(0,0) | 0.02(0,0.21) | 0.04(-0.01,0.39) | 2.21(-12.3,19.13) |
| East Asia | Papua New Guinea | 454(300,645) | 4.6(3.04,6.54) | 11.23(7.51,15.61) | 0.16(-0.62,0.95) |
| East Asia | Samoa | 2(0,6) | 0.86(0.04,3.04) | 1.38(0.07,4.84) | -0.29(-10.31,10.85) |
| East Asia | Tonga | 2 (1,4) | 2.03(0.6,4.21) | 2.7(0.79,5.6) | -1.02(-15.11,15.42) |
| East Asia | Vanuatu | 8(2,18) | 2.85(0.67,6.17) | 5.55(1.33,11.94) | -1.18(-6.6,4.54) |
| East Asia | Australia | 2572(1939,3199) | 10.47(7.89,13.02) | 5.37(4.07,6.67) | -4.37(-5.38,-3.36) |
| East Asia | Malaysia | 478(181,875) | 1.53(0.58,2.8) | 2.06(0.78,3.77) | -1.21(-2.15,-0.25) |
| Europe | Bosnia and Herzegovina | 1601 (1025,2251) | 48.52(31.07,68.21) | 29.04(18.5,40.77) | -2.94(-3.92,-1.95) |
| Europe | Kyrgyzstan | 1792(841,2947) | 27.42(12.87,45.09) | 47.91(21.82,79.85) | -2.03(-2.63,-1.42) |
| Europe | Republic of Moldova | 1900(737,3141) | 51.51(19.97,85.16) | 32.75(12.67,54.13) | -2.16(-3.15,-1.16) |
| Europe | Slovakia | 1969.17(1192.48,2822.8) | 36.22(21.93,51.92) | 21.9(13.23,31.4) | -3.73(-4.82,-2.64) |
| Europe | Turkey | 14289.57(10291.01,18668.83) | 17.56(12.65,22.95) | 17.62(12.72,22.95) | -3.84(-4.19,-3.49) |
| Europe | France | 11409.32(7182.57,15623.71) | 17.23(10.85,23.6) | 6.27(3.99,8.6) | -3.38(-3.98,-2.77) |
| Europe | Hungary | 4624.37(1729.89,7530.34) | 47.8(17.88,77.84) | 22.44(8.37,36.59) | -4.27(-5.06,-3.46) |
| Europe | United Kingdom | 13723.31(6776.14,21119.92) | 20.42(10.08,31.42) | 9.58(4.65,14.78) | -3.95(-4.68,-3.21) |
| Europe | Ukraine | 34007.01(21812.45,49277.08) | 77.21(49.53,111.89) | 44.34(28.35,64.24) | -0.06(-0.57,0.44) |
| Europe | San Marino | 6.94(4.18,10.51) | 20.96(12.64,31.76) | 8.45(5.01,13.02) | -1.46(-21.85,24.24) |
| Europe | Azerbaijan | 3504.1(2764.05,4285.61) | 34.09(26.89,41.69) | 57.72(45.82,70.74) | -2.03(-2.48,-1.59) |
| Europe | Iceland | 68.49(25.73,121.72) | 19.86(7.46,35.29) | 10.32(3.86,18.38) | -4.3(-9.22,0.88) |
| Europe | Georgia | 2559.16(1805.6,3496.14) | 69.83(49.27,95.4) | 39.04(27.49,53.33) | -2.89(-3.51,-2.28) |
| Europe | Poland | 12454.79(6746.8,19186.21) | 32.41(17.55,49.92) | 16.75(9.06,25.69) | -4.44(-4.94,-3.94) |
| Europe | Norway | 975.52(486.35,1594.19) | 18.24(9.09,29.8) | 8.54(4.26,14.13) | -5.51(-7.33,-3.66) |
| Europe | Monaco | 12.07(7.16,17.65) | 32.13(19.07,46.96) | 10.01(5.83,14.75) | -2.81(-18.64,16.11) |
| Europe | Uzbekistan | 9306.78(6408.47,12303.7) | 27.64(19.03,36.53) | 82.02(56.67,107.62) | 0.78(0.46,1.1) |
| Europe | Sweden | 2767.05(1523.67,4455.48) | 27.07(14.9,43.58) | 10.6(5.83,17.02) | -3.96(-5.27,-2.64) |
| Europe | Belgium | 2349.85(1157.83,3542.01) | 20.58(10.14,31.02) | 8.33(4.07,12.64) | -4.17(-5.14,-3.18) |
| Europe | Finland | 2049.04(783.19,3631.64) | 37.03(14.15,65.62) | 13.9(5.31,25.16) | -4.07(-5.46,-2.65) |
| Europe | Czechia | 3638.01(2053.53,5453.4) | 34.18(19.29,51.24) | 16.4(9.23,24.61) | -5.13(-6.17,-4.08) |
| Europe | Montenegro | 340.3(253.61,446.72) | 54.86(40.88,72.01) | 37.41(27.91,49.1) | -2.06(-4.54,0.48) |
| Europe | Italy | 18535.25(14442.6,22832.23) | 30.73(23.95,37.86) | 9.81(7.67,12.05) | -3.88(-4.66,-3.1) |
| Europe | Greece | 4599.95(3681.63,5498.1) | 44.5(35.62,53.19) | 15.26(12.18,18.23) | -2.27(-3.08,-1.45) |
| Europe | Netherlands | 2926.97(1253.63,4769.48) | 17.06(7.31,27.8) | 7.77(3.35,12.66) | -4.88(-5.75,-3.99) |
| Europe | Belarus | 5602.6(3071.14,8624.98) | 58.97(32.33,90.78) | 34.27(18.75,52.75) | -1.77(-2.49,-1.04) |
| Europe | Kazakhstan | 5171.42(3613.35,6982.85) | 28.12(19.65,37.97) | 38.2(26.72,51.29) | -2.86(-3.38,-2.34) |
| Europe | Spain | 9323.55(7171.15,11605.83) | 20.26(15.58,25.22) | 7.52(5.83,9.35) | -4.26(-4.93,-3.59) |
| Europe | Lithuania | 1586.96(688.58,2589.05) | 56.79(24.64,92.66) | 24.16(10.46,39.33) | -2.2(-3.62,-0.75) |
| Europe | Armenia | 1052.59(570.64,1644.14) | 34.86(18.9,54.45) | 26.88(14.42,41.98) | -2.85(-4.01,-1.69) |
| Europe | Romania | 12232.51(8366.68,16436.12) | 63.59(43.49,85.44) | 30.65(20.89,41.39) | -3.14(-3.71,-2.56) |
| Europe | Turkmenistan | 1555.84(1210.75,1988.52) | 30.61(23.82,39.12) | 47.82(37.36,60.54) | -1.11(-1.67,-0.55) |
| Europe | Albania | 1124.37(773.47,1520.18) | 41.33(28.43,55.88) | 27.29(18.8,36.98) | -1.18(-2.13,-0.22) |
| Europe | Croatia | 2027.81(1162.82,2956.84) | 47.74(27.37,69.61) | 21.66(12.34,31.71) | -4.09(-5.44,-2.73) |
| Europe | Portugal | 3055.03(2438.32,3698.45) | 28.68(22.89,34.72) | 10.39(8.32,12.52) | -5.64(-6.8,-4.47) |
| Europe | Switzerland | 1798.76(1050.66,2625.18) | 20.5(11.97,29.92) | 8(4.72,11.76) | -5.07(-6.67,-3.44) |
| Europe | North Macedonia | 1299(869.22,1771.64) | 60.34(40.38,82.3) | 52.25(34.9,70.05) | -2.55(-3.59,-1.5) |
| Europe | Austria | 2200.61(1245.43,3234.09) | 24.68(13.97,36.27) | 10.06(5.67,14.73) | -4.86(-6.09,-3.62) |
| Europe | Germany | 23760.14(12221.83,35229.94) | 27.98(14.39,41.49) | 10.35(5.28,15.44) | -4.44(-5.26,-3.62) |
| Europe | Andorra | 11.84(4.77,20.58) | 14.25(5.75,24.77) | 7.42(2.97,12.96) | -2.62(-15.83,12.67) |
| Europe | Serbia | 5448.12(3388.35,7842.55) | 62.29(38.74,89.66) | 37.97(23.56,54.24) | -3.87(-4.92,-2.81) |
| Europe | Greenland | 15.82(5.06,25.79) | 28.16(9.01,45.91) | 28.13(9.2,45.75) | -3.44(-11.14,4.92) |
| Europe | Ireland | 768.38(210.33,1366.62) | 15.65(4.28,27.83) | 9.84(2.68,17.51) | -5.05(-6.68,-3.39) |
| Europe | Latvia | 1227.34(673.26,1953.5) | 64.08(35.15,102) | 26.98(14.79,42.71) | -3.62(-5.49,-1.72) |
| Europe | Tajikistan | 2353.27(1316.79,3582.79) | 24.79(13.87,37.74) | 74.04(40.84,112.05) | -0.65(-1.22,-0.09) |
| Europe | Russian Federation | 86634.05(47053.07,142993.58) | 59.05(32.07,97.46) | 37(20.06,61.14) | -1.97(-2.55,-1.39) |
| Europe | Luxembourg | 89.32(38.41,148.94) | 14.44(6.21,24.08) | 7.77(3.31,12.91) | -5.31(-9.41,-1.02) |
| Europe | Slovenia | 529(335.18,756.29) | 25.5(16.16,36.46) | 10.27(6.49,14.69) | -5.34(-8.02,-2.59) |
| Europe | Cyprus | 182.61(111.94,273.86) | 13.9(8.52,20.85) | 11.22(6.91,16.88) | -4.06(-6.78,-1.27) |
| Europe | Bulgaria | 6936.15(4746.5,9469.51) | 100.02(68.45,136.55) | 47.08(31.88,64.42) | -2.37(-2.93,-1.81) |
| Europe | Estonia | 556.2(182.29,1024.21) | 42.38(13.89,78.04) | 18.13(6.04,33.32) | -5.23(-7.84,-2.54) |
| Europe | Denmark | 1079.29(433.13,1813.26) | 18.6(7.46,31.25) | 8.35(3.36,14.01) | -5.27(-7.13,-3.37) |
| Latin America | Antigua and Barbuda | 0.41(0,1.48) | 0.46(0,1.67) | 0.46(0,1.68) | -2.97(-31.7,37.84) |
| Latin America | Bahamas | 5.4(1.6,12.44) | 1.43(0.42,3.3) | 1.54(0.46,3.54) | -1.18(-8.63,6.88) |
| Latin America | Bolivia (Plurinational State of) | 784.35(539.79,1070.11) | 6.53(4.49,8.91) | 10.55(7.34,14.25) | -2.71(-3.36,-2.04) |
| Latin America | Saint Kitts and Nevis | 0.66(0,1.7) | 1.11(0,2.86) | 1.25(0,3.21) | -5.14(-32.85,34.01) |
| Latin America | Saint Lucia | 1.25(0.1,3.52) | 0.72(0.06,2.02) | 0.62(0.05,1.75) | -4.07(-30.23,31.88) |
| Latin America | Saint Vincent and the Grenadines | 1.33(0.14,3.57) | 1.18(0.12,3.16) | 1.1(0.12,2.94) | -2.87(-26.61,28.57) |
| Latin America | Trinidad and Tobago | 5.1(0,25.37) | 0.37(0,1.83) | 0.29(0,1.43) | -4.73(-11.89,3.02) |
| Latin America | United States Virgin Islands | 2.21(0.31,5.91) | 2.12(0.29,5.69) | 1.3(0.18,3.47) | -1.83(-29.28,36.29) |
| Latin America | Venezuela (Bolivarian Republic of) | 1104.82(651.64,1648.73) | 3.94(2.32,5.87) | 3.99(2.35,5.93) | -1.88(-2.57,-1.19) |
| Latin America | Grenada | 0.79(0.08,2.2) | 0.76(0.08,2.14) | 0.81(0.09,2.23) | -3.45(-32.28,37.64) |
| Latin America | Guatemala | 469.54(328.64,633.86) | 2.64(1.85,3.57) | 5.17(3.68,6.89) | -3.21(-3.95,-2.47) |
| Latin America | Panama | 58.19(29.33,94.82) | 1.4(0.7,2.28) | 1.35(0.69,2.21) | -2.04(-5.41,1.45) |
| Latin America | Uruguay | 683.59(520.91,856.65) | 19.89(15.16,24.93) | 11.01(8.44,13.8) | -3.32(-4.69,-1.94) |
| Latin America | Nicaragua | 78.53(27.71,144) | 1.21(0.43,2.21) | 2.28(0.81,4.19) | -0.59(-3.25,2.15) |
| Latin America | Ecuador | 905.89(660.58,1182.52) | 5.15(3.76,6.72) | 6.91(5.08,8.96) | -1.73(-2.34,-1.12) |
| Latin America | Brazil | 8701.79(5853.7,12095.77) | 4.02(2.7,5.58) | 3.84(2.58,5.34) | -3.77(-4,-3.54) |
| Latin America | Colombia | 2210.28(1498.26,2968.66) | 4.63(3.14,6.21) | 3.99(2.71,5.37) | -3.64(-4.11,-3.17) |
| Latin America | Cuba | 284.05(108.37,563.44) | 2.5(0.95,4.96) | 1.4(0.53,2.8) | -2.34(-4.26,-0.39) |
| Latin America | Belize | 3.51(1.56,6.17) | 0.86(0.38,1.5) | 1.42(0.63,2.49) | -2.43(-12.66,8.99) |
| Latin America | Jamaica | 15.2(0.39,56.8) | 0.54(0.01,2.02) | 0.47(0.01,1.76) | -1.82(-7.6,4.33) |
| Latin America | Chile | 2631.26(1904.87,3500.82) | 14.46(10.47,19.24) | 11.07(8.01,14.75) | -2.29(-2.9,-1.68) |
| Latin America | El Salvador | 43.9(13.96,89.37) | 0.7(0.22,1.43) | 0.68(0.22,1.39) | -2.6(-5.35,0.22) |
| Latin America | Suriname | 5.25(0.87,13.74) | 0.91(0.15,2.39) | 0.93(0.15,2.43) | -0.55(-11.5,11.76) |
| Latin America | Dominican Republic | 496.15(272.46,778.13) | 4.56(2.5,7.15) | 5.69(3.15,8.86) | 1.24(0.24,2.24) |
| Latin America | Mexico | 9150.34(6873.85,11712.33) | 7.32(5.5,9.37) | 8.59(6.46,10.96) | -1.15(-1.4,-0.9) |
| Latin America | Haiti | 209.76(55.86,469.97) | 1.69(0.45,3.79) | 3.66(1,8.13) | -0.73(-2.08,0.63) |
| Latin America | Dominica | 1.6(0.15,4.75) | 2.32(0.21,6.92) | 1.76(0.16,5.23) | -2.01(-27.88,33.14) |
| Latin America | Costa Rica | 184.88(119.99,264.3) | 3.92(2.54,5.6) | 3.56(2.31,5.09) | -2.06(-4.06,-0.01) |
| Latin America | Guyana | 23.08(9.65,41.59) | 2.99(1.25,5.4) | 4.27(1.79,7.6) | -1.67(-5.58,2.41) |
| Latin America | Honduras | 350.31(160.61,618.3) | 3.57(1.64,6.3) | 7(3.27,12.35) | -0.88(-2.1,0.34) |
| Latin America | Argentina | 6847.68(5314.18,8544.1) | 15.18(11.78,18.94) | 12.39(9.64,15.43) | -3.52(-3.82,-3.23) |
| Latin America | Peru | 1755.63(1182,2478.58) | 5.16(3.48,7.29) | 5.33(3.57,7.54) | -3.02(-3.44,-2.6) |
| Latin America | Paraguay | 311.2(160.51,499.01) | 4.49(2.32,7.2) | 5.9(3.05,9.47) | -1.8(-3.28,-0.3) |
| Latin America | Barbados | 2.95(0.29,8.08) | 0.99(0.1,2.71) | 0.6(0.06,1.65) | -3.33(-25.28,25.07) |
| Latin America | Puerto Rico | 64.74(12.78,177.62) | 1.84(0.36,5.04) | 0.79(0.16,2.2) | -3.63(-8.64,1.66) |
| Middle East | Egypt | 15329.56(8297.93,23679.65) | 15.47(8.38,23.9) | 30.37(16.74,46.55) | -0.87(-1.03,-0.7) |
| Middle East | Iran (Islamic Republic of) | 13608.15(10928.85,16305.81) | 16.14(12.96,19.34) | 21.85(17.52,26.02) | -3.31(-3.5,-3.12) |
| Middle East | Palestine | 446.74(259.85,647.54) | 9.01(5.24,13.06) | 25.34(14.85,36.94) | -2.9(-4.03,-1.75) |
| Middle East | Yemen | 1882.93(897.82,2857.53) | 5.98(2.85,9.07) | 17.46(8.45,25.82) | -1.55(-2,-1.11) |
| Middle East | Djibouti | 25.68(0.81,48.26) | 2.14(0.07,4.01) | 6(0.23,10.98) | -0.38(-3.97,3.34) |
| Middle East | Kuwait | 243.5(45.73,434.17) | 5.5(1.03,9.81) | 10.7(1.97,19.08) | -2.06(-3.33,-0.76) |
| Middle East | Jordan | 883.15(581.8,1248.3) | 7.59(5,10.73) | 18.55(12.28,25.89) | -3.8(-4.65,-2.94) |
| Middle East | Libya | 721.27(363.8,1121.09) | 10.71(5.4,16.64) | 15.96(8.02,24.82) | -0.57(-1.3,0.16) |
| Middle East | Oman | 223(9.48,436.44) | 4.86(0.21,9.52) | 21.75(0.94,42) | -3.57(-4.67,-2.47) |
| Middle East | Morocco | 8937.44(6692.51,11233.36) | 24.86(18.62,31.25) | 35.09(26.54,43.97) | -1.83(-2.09,-1.57) |
| Middle East | Qatar | 59.55(0,120.12) | 2.08(0,4.19) | 18.08(0,35.28) | -4.87(-7.11,-2.58) |
| Middle East | Tunisia | 2576.61(1610.44,3734.38) | 22.27(13.92,32.27) | 23.37(14.62,33.71) | -1.57(-2.14,-1) |
| Middle East | Iraq | 5151.6(3166.66,7204.08) | 12.23(7.52,17.1) | 28.22(17.3,38.8) | -2.38(-2.68,-2.09) |
| Middle East | Israel | 650.41(349.7,995.01) | 6.99(3.76,10.69) | 5.07(2.72,7.74) | -6.44(-7.99,-4.86) |
| Middle East | Lebanon | 1311.12(834.19,1777.97) | 25.33(16.11,34.34) | 25.88(16.44,35.15) | -2.02(-2.72,-1.32) |
| Middle East | Malta | 96.23(35.02,169.7) | 21.91(7.97,38.64) | 9.46(3.46,16.66) | -3.37(-7.88,1.37) |
| Middle East | Syrian Arab Republic | 3617.87(2417.89,5127.09) | 24.97(16.69,35.38) | 38.9(26.39,53.52) | -2.57(-2.85,-2.28) |
| Middle East | United Arab Emirates | 453.84(0,975.96) | 4.91(0,10.56) | 17.13(-0.02,35.91) | -2.34(-3.29,-1.38) |
| Middle East | Bahrain | 52.32(12.35,91.21) | 3.63(0.86,6.32) | 9.68(2.47,16.93) | -5.69(-7.92,-3.39) |
| Middle East | Algeria | 5817.75(3459.46,8238.83) | 13.9(8.27,19.69) | 23.64(14.09,33.21) | -3.2(-3.49,-2.91) |
| Middle East | Saudi Arabia | 2279.32(1062.1,3479.38) | 6.38(2.97,9.74) | 16.32(7.68,24.79) | -1.05(-1.45,-0.65) |
| North America | United States of America | 66378.05(52143.11,80903.89) | 20.24(15.9,24.67) | 10.86(8.62,13.19) | -2.42(-2.82,-2.02) |
| North America | Bermuda | 7.92(0,18.7) | 12.37(0,29.2) | 5.64(0,13.34) | -4.5(-20.45,14.64) |
| North America | Canada | 7935.2(2707.17,13284.42) | 21.73(7.41,36.38) | 10.31(3.56,17.23) | -3.44(-4,-2.88) |
| South Asia | Bangladesh | 7305.37(3136.41,12031.65) | 4.59(1.97,7.55) | 6.34(2.67,10.37) | -1(-1.37,-0.63) |
| South Asia | Pakistan | 18340.47(8771.98,28004.71) | 8.19(3.91,12.5) | 19.45(9.39,29.37) | 0.32(0.19,0.46) |
| South Asia | Afghanistan | 4934.06(3485.73,6604.29) | 12.89(9.11,17.25) | 46.95(33.6,61.31) | -1.83(-2.04,-1.61) |
| South Asia | Nepal | 3562(2517.57,4780.83) | 11.71(8.28,15.72) | 18.83(13.32,24.97) | -1.23(-1.63,-0.84) |
| South Asia | Bhutan | 105.04(71.44,144.12) | 13.93(9.47,19.11) | 21.52(14.66,29.61) | -1.78(-3.87,0.36) |
| South Asia | Sri Lanka | 373.74(156.88,604.48) | 1.71(0.72,2.77) | 1.71(0.72,2.75) | -2.56(-3.68,-1.43) |
| South Asia | Maldives | 0.02(-0.01,0.15) | 0(0,0.03) | 0.01(0,0.06) | -6.27(-20.99,11.19) |
| South Asia | India | 73282.26(36147.33,110735) | 5.27(2.6,7.96) | 7.25(3.62,10.96) | -0.88(-1.12,-0.64) |
| Sub Saharan Africa | Congo | 70.9(15.66,153.26) | 1.35(0.3,2.91) | 3.6(0.83,7.73) | -3.01(-5.03,-0.94) |
| Sub Saharan Africa | Coate d'Ivoire | 88.48(32.71,178.03) | 0.34(0.12,0.68) | 1.1(0.4,2.19) | -2.33(-3.94,-0.69) |
| Sub Saharan Africa | Democratic Republic of the Congo | 2125.9(1238.42,3258.85) | 2.42(1.41,3.72) | 7.71(4.55,11.79) | -1.1(-1.51,-0.69) |
| Sub Saharan Africa | Gambia | 20.71(3.13,34.85) | 0.92(0.14,1.55) | 2.57(0.39,4.25) | 0.39(-5.38,6.51) |
| Sub Saharan Africa | Sao Tome and Principe | 1.02(-0.01,3.93) | 0.5(0,1.92) | 1.23(0,4.71) | -1.31(-18.92,20.11) |
| Sub Saharan Africa | United Republic of Tanzania | 1709.84(985.27,2688.73) | 3.01(1.74,4.74) | 8.61(5.06,13.35) | -1.39(-1.87,-0.9) |
| Sub Saharan Africa | Zambia | 622.6(249.72,1165.11) | 3.41(1.37,6.39) | 11.87(4.9,21.72) | -0.23(-0.95,0.5) |
| Sub Saharan Africa | Botswana | 131.05(53.41,240.94) | 5.6(2.28,10.3) | 12.52(5.06,22.6) | -0.71(-2.44,1.05) |
| Sub Saharan Africa | Comoros | 7.44(0.31,23.46) | 1.04(0.04,3.28) | 1.77(0.08,5.57) | -2.23(-8.87,4.9) |
| Sub Saharan Africa | Guinea-Bissau | 13.54(0.34,25.55) | 0.71(0.02,1.34) | 2.33(0.06,4.31) | 0.35(-3.18,4) |
| Sub Saharan Africa | Ethiopia | 3114.52(2293.54,4124.05) | 2.89(2.13,3.83) | 9.38(6.84,12.35) | -3.93(-4.22,-3.63) |
| Sub Saharan Africa | South Sudan | 37.46(11.09,66.07) | 0.4(0.12,0.71) | 1.26(0.38,2.2) | -3.62(-5.87,-1.32) |
| Sub Saharan Africa | Lesotho | 353.62(231.02,498.65) | 16.91(11.05,23.84) | 35.41(23.52,49.31) | 2.09(0.85,3.35) |
| Sub Saharan Africa | Niger | 578.24(37.2,1131.85) | 2.48(0.16,4.86) | 9.69(0.68,18.88) | -1.49(-2.35,-0.63) |
| Sub Saharan Africa | Benin | 57.67(2,108.34) | 0.46(0.02,0.86) | 1.45(0.05,2.66) | -2.75(-5.24,-0.21) |
| Sub Saharan Africa | Eswatini | 90.53(57.59,136.43) | 7.93(5.04,11.95) | 20.46(13.1,30.78) | 0.67(-1.46,2.85) |
| Sub Saharan Africa | Senegal | 167.44(13.65,294.72) | 1.11(0.09,1.95) | 2.72(0.22,4.72) | 0.38(-1.16,1.96) |
| Sub Saharan Africa | Cabo Verde | 37.12(8.06,78.43) | 6.59(1.43,13.92) | 9.01(1.95,19.11) | -0.89(-4.95,3.34) |
| Sub Saharan Africa | Seychelles | 0.46(0.01,1.63) | 0.45(0.01,1.6) | 0.47(0.01,1.68) | -3.02(-23.98,23.71) |
| Sub Saharan Africa | Chad | 421.7(107.86,766.58) | 2.57(0.66,4.67) | 9.08(2.39,16.18) | -0.68(-1.63,0.27) |
| Sub Saharan Africa | Uganda | 655.58(307.38,1121.98) | 1.59(0.75,2.73) | 5.92(2.86,10.07) | -1.44(-2.13,-0.74) |
| Sub Saharan Africa | Kenya | 1602.81(1129.84,2195.27) | 3.19(2.25,4.37) | 9.64(6.86,13.09) | -0.08(-0.59,0.44) |
| Sub Saharan Africa | South Africa | 5404.74(4532.48,6372.55) | 9.72(8.15,11.46) | 14.57(12.22,17.29) | -2.32(-2.82,-1.81) |
| Sub Saharan Africa | Ghana | 114.71(18.9,224.08) | 0.36(0.06,0.71) | 0.92(0.15,1.78) | -3.41(-4.89,-1.92) |
| Sub Saharan Africa | Mauritius | 75.53(0,201.01) | 5.92(0,15.74) | 4.75(0,12.59) | -4.8(-7.44,-2.08) |
| Sub Saharan Africa | Liberia | 22.71(2.69,67.02) | 0.47(0.06,1.4) | 1.39(0.16,4.14) | -1.73(-5.38,2.06) |
| Sub Saharan Africa | Sierra Leone | 38.06(9.78,90.34) | 0.46(0.12,1.09) | 1.27(0.34,2.99) | -0.91(-3.5,1.75) |
| Sub Saharan Africa | Mauritania | 167.65(20.14,312.33) | 4.18(0.5,7.78) | 9.6(1.22,17.71) | -2.17(-3.97,-0.34) |
| Sub Saharan Africa | Somalia | 201.73(102.73,316.19) | 0.99(0.51,1.55) | 3.96(2.07,6.07) | -0.7(-1.8,0.42) |
| Sub Saharan Africa | Cameroon | 412.75(178.76,749.69) | 1.42(0.61,2.58) | 4.37(1.97,7.87) | -0.61(-1.53,0.31) |
| Sub Saharan Africa | Burundi | 526.57(259.5,866.03) | 4.41(2.17,7.26) | 14.87(7.51,24.28) | -2.35(-3.06,-1.63) |
| Sub Saharan Africa | Eritrea | 124.35(57.83,196.97) | 1.85(0.86,2.94) | 6.26(2.98,9.65) | -2.44(-3.73,-1.13) |
| Sub Saharan Africa | Malawi | 597.5(349.06,921.75) | 3.24(1.89,5) | 10.08(5.89,15.34) | -0.85(-1.59,-0.11) |
| Sub Saharan Africa | Namibia | 154.02(86.45,245.45) | 6.41(3.6,10.21) | 13.05(7.33,20.47) | -1.54(-3.47,0.42) |
| Sub Saharan Africa | Rwanda | 675.78(423.03,999.8) | 5.33(3.33,7.88) | 14.98(9.54,21.72) | -4.37(-5,-3.74) |
| Sub Saharan Africa | Sudan | 2676.87(1025.97,4398.18) | 6.56(2.51,10.78) | 16.98(6.6,27.55) | -2.56(-2.88,-2.24) |
| Sub Saharan Africa | Togo | 27.89(7.86,52.43) | 0.35(0.1,0.66) | 1(0.29,1.86) | -2.94(-5.86,0.08) |
| Sub Saharan Africa | Guinea | 104.73(45.66,184.83) | 0.83(0.36,1.46) | 2.18(0.95,3.87) | 0.2(-1.76,2.2) |
| Sub Saharan Africa | Central African Republic | 52.79(22.21,97.19) | 1(0.42,1.83) | 3.13(1.35,5.66) | -1.46(-3.43,0.56) |
| Sub Saharan Africa | Mali | 685.19(-13.51,1440.97) | 3.13(-0.06,6.57) | 10.06(-0.02,21) | -1.32(-2.1,-0.53) |
| Sub Saharan Africa | Madagascar | 1172.47(734.96,1719.74) | 4.39(2.75,6.44) | 13.8(8.84,19.75) | -1.16(-1.6,-0.71) |
| Sub Saharan Africa | Zimbabwe | 721.2(427.71,1115.27) | 4.8(2.85,7.43) | 13.41(8.13,20.57) | 2.24(1.36,3.12) |
| Sub Saharan Africa | Gabon | 23.21(5.92,48.66) | 1.33(0.34,2.78) | 2.78(0.71,5.83) | -3.01(-6.77,0.9) |
| Sub Saharan Africa | Equatorial Guinea | 13.94(2.36,34.95) | 0.98(0.17,2.46) | 3.88(0.69,9.66) | -5.15(-9.68,-0.4) |
| Sub Saharan Africa | Mozambique | 731.02(333.19,1242.68) | 2.48(1.13,4.21) | 8.3(3.95,14.04) | 1.83(1.04,2.62) |
| Sub Saharan Africa | Burkina Faso | 272.8(0.29,497.61) | 1.2(0,2.19) | 3.67(0,6.62) | -0.72(-1.99,0.56) |
| Sub Saharan Africa | Nigeria | 804.68(107.84,1813.35) | 0.37(0.05,0.84) | 1.13(0.16,2.53) | -3.83(-4.47,-3.18) |
| Sub Saharan Africa | Angola | 734.71(417.99,1166.59) | 2.44(1.39,3.87) | 8.94(5.22,14.06) | -1.77(-2.43,-1.12) |

# Table S3. Number of DALYs, all-age DALYs, age-standardized DALYs and net drift of DALYs attributed to low temperature distribution in 204 countries and territories.

| **Regions** | **Countries** | **Number in 2019** | **All-age DALYs rate**  **per 100,000 in 2019** | **Age-standardized DALYs rate** | **Net drift of DALYs, % per year** |
| --- | --- | --- | --- | --- | --- |
| East Asia | Cook Islands | 19.46(2.41,46.41) | 108.21(13.41,258.03) | 81.88(10.23,197.27) | -1.92(-5.26,1.54) |
| East Asia | Democratic People's Republic of Korea | 202191.64(135810.66,287989.53) | 770.76(517.71,1097.82) | 639.54(431.09,906.82) | -0.09(-0.14,-0.03) |
| East Asia | Lao People's Democratic Republic | 8218.17(3868.36,14043.01) | 114.81(54.04,196.18) | 186.82(89.22,317.6) | -1.56(-1.69,-1.43) |
| East Asia | Micronesia (Federated States of) | 0.4(-0.04,2.48) | 0.4(-0.04,2.43) | 0.53(-0.06,3.33) | -2.37(-4.61,-0.08) |
| East Asia | Niue | 1.23(0.09,3.52) | 73.55(5.3,210.67) | 57.83(4.06,166.51) | -2.67(-14.16,10.37) |
| East Asia | Republic of Korea | 102755.2(70775.95,140118.93) | 192.43(132.54,262.4) | 120.86(83.42,165.34) | -7.14(-7.42,-6.85) |
| East Asia | Taiwan (Province of China) | 32629.96(22446.18,46085.2) | 138.14(95.03,195.11) | 84.35(57.89,119.21) | -3.15(-3.44,-2.85) |
| East Asia | Tokelau | 0.21(0,0.46) | 15.14(0,32.88) | 16.36(0,35.5) | -1.24(-26.35,32.42) |
| East Asia | Viet Nam | 111436.57(51129.8,193753.04) | 115.63(53.05,201.05) | 124.57(57.26,216.83) | -1.03(-1.1,-0.96) |
| East Asia | Brunei Darussalam | 22.37(1.96,64.92) | 5.12(0.45,14.85) | 7.92(0.69,23.12) | -4.66(-7.4,-1.83) |
| East Asia | China | 7003989.08(5554314.62,8713803.55) | 492.42(390.5,612.63) | 374.02(298.15,464.32) | -1.95(-2.12,-1.78) |
| East Asia | Mongolia | 24684.32(11817.32,41315.82) | 728.67(348.84,1219.62) | 1042.64(510.7,1750.74) | -1.24(-1.43,-1.05) |
| East Asia | Guam | 31.15(0,65.3) | 18.26(0,38.27) | 16.5(0,34.52) | -3.39(-5.75,-0.97) |
| East Asia | Nauru | 0.83(0,2.33) | 7.83(0,22.13) | 15.1(0,42.68) | -2.19(-10.71,7.15) |
| East Asia | Northern Mariana Islands | 11.62(2.02,21.32) | 27.34(4.75,50.17) | 21.64(3.66,39.32) | -1.58(-5.38,2.38) |
| East Asia | Palau | 0.13(-0.51,1.13) | 0.72(-2.84,6.26) | 0.57(-2.35,5.01) | -2.68(-8.96,4.04) |
| East Asia | Timor-Leste | 640.15(191.9,1291.78) | 47.96(14.38,96.78) | 81.13(24.87,163.84) | 1(0.37,1.63) |
| East Asia | Singapore | 455.63(0,877.86) | 8.04(0,15.49) | 5.87(0,11.3) | -5.03(-6.06,-3.99) |
| East Asia | Fiji | 925.87(310.03,1761.66) | 101.6(34.02,193.32) | 120.56(40.91,228.22) | -2.18(-2.56,-1.8) |
| East Asia | Cambodia | 1324.64(474.47,2366.14) | 7.98(2.86,14.25) | 11.44(4.07,20.53) | -4.2(-4.42,-3.97) |
| East Asia | Myanmar | 98877.04(63496.5,143961.13) | 180.84(116.13,263.29) | 217.29(139.82,314.05) | -3.07(-3.16,-2.98) |
| East Asia | Thailand | 18101.47(7647.18,33522.93) | 25.82(10.91,47.81) | 18.38(7.75,34.07) | -3.85(-4.12,-3.58) |
| East Asia | Indonesia | 151081.86(69141.01,263909.47) | 58.23(26.65,101.71) | 71.06(31.88,125.93) | -0.67(-0.72,-0.63) |
| East Asia | Marshall Islands | 0.16(-0.04,1.11) | 0.28(-0.07,1.95) | 0.41(-0.1,2.84) | -1.34(-4.72,2.15) |
| East Asia | Solomon Islands | 152.58(30.43,393.82) | 23.27(4.64,60.07) | 41.45(8.17,106.88) | -2.18(-2.81,-1.55) |
| East Asia | Japan | 324958.07(254186,387444.4) | 254.29(198.91,303.19) | 92.02(75.43,108.16) | -2.63(-2.98,-2.29) |
| East Asia | Tuvalu | 1.88(0,4.11) | 15.94(0,34.84) | 18.29(0,39.63) | -1.58(-10.07,7.7) |
| East Asia | New Zealand | 11389.22(8202.34,14798.14) | 253.34(182.45,329.16) | 144.03(104.1,186.96) | -4.6(-4.93,-4.27) |
| East Asia | American Samoa | 9.8(0,19.26) | 17.65(0,34.71) | 20.22(0,40.02) | -0.92(-4.85,3.18) |
| East Asia | Philippines | 20527.21(8610.54,35025.73) | 18.3(7.68,31.23) | 25.34(10.85,43.05) | 3.68(3.4,3.96) |
| East Asia | Kiribati | 0.85(-0.1,7.79) | 0.71(-0.08,6.57) | 1.05(-0.12,9.61) | 2.23(-0.1,4.61) |
| East Asia | Papua New Guinea | 13274.44(8717.99,19143.69) | 134.54(88.36,194.02) | 246.13(162.92,349.69) | 0.17(0.06,0.29) |
| East Asia | Samoa | 43.06(2.19,152.75) | 20.37(1.03,72.27) | 28.71(1.45,101.47) | -0.25(-1.73,1.24) |
| East Asia | Tonga | 43.35(12.65,91.34) | 42.36(12.35,89.25) | 54.22(15.89,114.19) | -1.01(-3.07,1.11) |
| East Asia | Vanuatu | 227.13(54.12,494.65) | 77.11(18.37,167.93) | 123.07(29.14,268.02) | -1.17(-1.97,-0.37) |
| East Asia | Australia | 32824.07(24997.55,40624.47) | 133.6(101.75,165.35) | 76.79(58.53,94.35) | -4.29(-4.74,-3.84) |
| East Asia | Malaysia | 10580.37(3976.07,19599.88) | 33.8(12.7,62.62) | 39.69(15.06,73.13) | -1.24(-1.42,-1.06) |
| Europe | Bosnia and Herzegovina | 25022.79(15807.98,35334.09) | 758.27(479.03,1070.74) | 435.51(273.36,616.25) | -2.94(-3.11,-2.78) |
| Europe | Kyrgyzstan | 35784.46(18057.63,56839.49) | 547.54(276.3,869.71) | 826.6(410.96,1335.8) | -1.98(-2.25,-1.72) |
| Europe | Republic of Moldova | 32159.69(13181.08,53018.34) | 871.96(357.39,1437.52) | 557.37(227.86,920.18) | -2.12(-2.41,-1.82) |
| Europe | Slovakia | 30450.5(18478.26,43948.27) | 560.04(339.85,808.29) | 335.74(203.69,484.89) | -3.72(-3.99,-3.44) |
| Europe | Turkey | 245081.64(173912.37,323557.66) | 301.23(213.76,397.69) | 285.98(203.33,376.92) | -3.83(-4.09,-3.56) |
| Europe | France | 133935.18(84750.82,183305.08) | 202.31(128.01,276.88) | 92.22(57.72,126.56) | -3.33(-3.63,-3.02) |
| Europe | Hungary | 69030.32(25537.94,114745.41) | 713.54(263.97,1186.07) | 358.44(131.79,597.5) | -4.27(-4.48,-4.07) |
| Europe | United Kingdom | 185046.44(86728.22,287876.69) | 275.28(129.02,428.26) | 146.36(67.66,228.37) | -3.91(-4.25,-3.56) |
| Europe | Ukraine | 585038.82(374937.1,857500.35) | 1328.35(851.31,1946.99) | 781.05(499.74,1144.2) | -0.06(-0.39,0.27) |
| Europe | San Marino | 83.03(47.34,130.85) | 250.85(143.01,395.32) | 119.14(65.67,190.41) | -1.44(-4.44,1.64) |
| Europe | Azerbaijan | 71333.7(55594.62,88594.19) | 694(540.87,861.92) | 900.71(707.38,1101.82) | -2.01(-2.2,-1.82) |
| Europe | Iceland | 867.46(318.82,1549.33) | 251.53(92.45,449.24) | 149.9(54.19,268.95) | -4.29(-4.97,-3.62) |
| Europe | Georgia | 41204.87(28912.84,56507.96) | 1124.36(788.94,1541.93) | 687.9(481.58,944.51) | -2.86(-3.22,-2.5) |
| Europe | Poland | 183405.2(99463.62,279480.86) | 477.19(258.79,727.16) | 262.5(143.78,399.37) | -4.44(-4.68,-4.21) |
| Europe | Norway | 12024.53(5878.76,20200.41) | 224.81(109.91,377.66) | 118.84(57.67,199.47) | -5.48(-5.93,-5.03) |
| Europe | Monaco | 146.26(83.37,217.03) | 389.28(221.88,577.63) | 146.81(82.33,222.3) | -2.76(-4.99,-0.48) |
| Europe | Uzbekistan | 223179.18(154621.86,298281.24) | 662.7(459.13,885.71) | 1329.02(914.3,1751.02) | 0.77(0.56,0.99) |
| Europe | Sweden | 32733.19(17949.33,52300.45) | 320.21(175.59,511.62) | 144.39(79.16,231.47) | -3.91(-4.43,-3.38) |
| Europe | Belgium | 29591.7(14321.94,45116.74) | 259.14(125.42,395.1) | 123.05(58.8,188.09) | -4.14(-4.44,-3.83) |
| Europe | Finland | 25405.7(9808.49,46360.59) | 459.08(177.24,837.73) | 198.08(77.09,363.97) | -4.02(-4.5,-3.54) |
| Europe | Czechia | 49991.81(28251.31,74841.36) | 469.69(265.43,703.17) | 234.72(132.35,352.88) | -5.12(-5.47,-4.78) |
| Europe | Montenegro | 5534.22(4118.51,7305.45) | 892.13(663.91,1177.65) | 576.99(429.28,764.54) | -2.05(-2.38,-1.72) |
| Europe | Italy | 210345.29(165358.87,256858.56) | 348.76(274.17,425.87) | 132.38(104.73,161.91) | -3.85(-4.19,-3.51) |
| Europe | Greece | 58116.46(46774.13,69381.75) | 562.21(452.48,671.19) | 235.75(188.54,283.3) | -2.23(-2.51,-1.95) |
| Europe | Netherlands | 38473.82(16125.44,63176.47) | 224.25(93.99,368.23) | 111.37(45.74,183) | -4.82(-5.24,-4.39) |
| Europe | Belarus | 93335.03(51007.78,143226.1) | 982.39(536.88,1507.52) | 588.49(321.64,902.18) | -1.7(-2.08,-1.32) |
| Europe | Kazakhstan | 100490.73(69947.93,137907.01) | 546.38(380.32,749.82) | 629.08(437.07,853.52) | -2.78(-3.14,-2.42) |
| Europe | Spain | 112469.51(86728.04,139259.73) | 244.39(188.45,302.6) | 109.4(84.66,136.19) | -4.25(-4.56,-3.92) |
| Europe | Lithuania | 22354.21(10028.12,36160.18) | 800.02(358.89,1294.11) | 378.41(169.7,611.41) | -2.16(-2.65,-1.66) |
| Europe | Armenia | 17478.65(9455.46,27645.08) | 578.83(313.13,915.5) | 432.41(233.07,684.41) | -2.84(-3.2,-2.48) |
| Europe | Romania | 182368.86(122768.4,247775.8) | 948.01(638.19,1288.01) | 485.11(323.48,661.67) | -3.11(-3.41,-2.8) |
| Europe | Turkmenistan | 34606.19(26636.5,44535.79) | 680.81(524.02,876.16) | 908.96(703.33,1161.89) | -1.06(-1.28,-0.85) |
| Europe | Albania | 17287.74(11757.19,23832.87) | 635.5(432.19,876.09) | 418.48(284.07,575.57) | -1.13(-1.33,-0.94) |
| Europe | Croatia | 28049.08(15680.22,41387.33) | 660.3(369.13,974.3) | 311.93(174.22,462.31) | -4.08(-4.53,-3.63) |
| Europe | Portugal | 38037.34(30793.52,45694.27) | 357.12(289.11,429) | 149.37(121.18,179.27) | -5.63(-5.96,-5.3) |
| Europe | Switzerland | 20199.46(11951.51,29889.92) | 230.19(136.2,340.62) | 104.29(61.36,156.22) | -5.05(-5.47,-4.63) |
| Europe | North Macedonia | 22000.25(14471.76,30314.32) | 1021.97(672.25,1408.18) | 762.93(507.17,1047.94) | -2.52(-2.79,-2.26) |
| Europe | Austria | 26596.87(15089.83,39588.51) | 298.3(169.24,444.01) | 138.54(78.89,207.12) | -4.84(-5.14,-4.53) |
| Europe | Germany | 296259.21(148665.3,447246.09) | 348.89(175.08,526.7) | 146.76(72.89,222.56) | -4.43(-4.84,-4.03) |
| Europe | Andorra | 158.17(60.75,280.56) | 190.42(73.14,337.76) | 109.04(41.58,196.07) | -2.56(-4.41,-0.69) |
| Europe | Serbia | 81996.17(50017.47,118831.71) | 937.44(571.84,1358.58) | 534.11(325.87,772.95) | -3.83(-4.46,-3.2) |
| Europe | Greenland | 327.35(105.78,542.61) | 582.6(188.27,965.7) | 494.64(160.21,811.93) | -3.45(-4.48,-2.41) |
| Europe | Ireland | 10542.52(2675.12,19088.74) | 214.7(54.48,388.74) | 140.2(35.44,254.04) | -4.98(-5.44,-4.51) |
| Europe | Latvia | 17489.87(9475.59,27452.95) | 913.17(494.73,1433.36) | 423.74(229.53,665.21) | -3.6(-4.12,-3.07) |
| Europe | Tajikistan | 52715.98(29271.08,81014.7) | 555.35(308.36,853.47) | 1247.99(699.73,1893.82) | -0.7(-0.96,-0.43) |
| Europe | Russian Federation | 1471259.04(788354.47,2479252.6) | 1002.78(537.33,1689.81) | 637.29(342.37,1070.82) | -1.91(-2.24,-1.57) |
| Europe | Luxembourg | 1150.14(464.79,1927.08) | 185.94(75.14,311.55) | 110.09(43.83,183.93) | -5.3(-5.86,-4.75) |
| Europe | Slovenia | 6782.44(4228.33,9844.32) | 326.98(203.85,474.59) | 147.47(90.93,216.77) | -5.3(-5.81,-4.8) |
| Europe | Cyprus | 2853.04(1695.27,4325.99) | 217.21(129.07,329.35) | 159.33(95.63,240.06) | -4.04(-4.46,-3.63) |
| Europe | Bulgaria | 108793.27(73584.36,150740.87) | 1568.84(1061.12,2173.74) | 766.15(512.3,1069.84) | -2.36(-2.6,-2.12) |
| Europe | Estonia | 7575.02(2663.22,13843.94) | 577.21(202.93,1054.89) | 274.03(97.12,502.53) | -5.2(-5.7,-4.71) |
| Europe | Denmark | 14127.79(5685.73,23816.96) | 243.47(97.98,410.44) | 119.94(48.76,203.94) | -5.25(-5.61,-4.88) |
| Latin America | Antigua and Barbuda | 7.42(0,27.32) | 8.38(0,30.88) | 7.61(0,27.86) | -2.99(-7.45,1.7) |
| Latin America | Bahamas | 117.77(35.11,270.18) | 31.24(9.32,71.68) | 29.92(8.89,69.27) | -1.18(-2.24,-0.12) |
| Latin America | Bolivia (Plurinational State of) | 15915.85(10669.52,22108.87) | 132.5(88.83,184.06) | 184.5(124.89,254.41) | -2.69(-2.78,-2.59) |
| Latin America | Saint Kitts and Nevis | 13.61(0,34.58) | 22.87(0,58.11) | 21.7(0,55.56) | -5.16(-9.48,-0.63) |
| Latin America | Saint Lucia | 22.36(1.76,62.62) | 12.81(1.01,35.86) | 10.57(0.83,29.62) | -4.04(-8.03,0.13) |
| Latin America | Saint Vincent and the Grenadines | 24.28(2.3,63.92) | 21.46(2.03,56.49) | 18.58(1.79,49.16) | -2.83(-6.44,0.92) |
| Latin America | Trinidad and Tobago | 99.05(0,488.54) | 7.14(0,35.21) | 5.41(0,26.86) | -4.72(-5.69,-3.74) |
| Latin America | United States Virgin Islands | 41.12(5.91,109.69) | 39.54(5.68,105.49) | 22.98(3.33,61.21) | -1.86(-5.94,2.41) |
| Latin America | Venezuela (Bolivarian Republic of) | 21757.12(12480.28,32959.25) | 77.51(44.46,117.42) | 75.08(43.03,113.24) | -1.89(-2.03,-1.74) |
| Latin America | Grenada | 15.8(1.7,43.98) | 15.31(1.64,42.61) | 14.5(1.55,40.48) | -3.47(-7.92,1.19) |
| Latin America | Guatemala | 9349.11(6477.48,12930.15) | 52.59(36.44,72.74) | 83.59(58.34,114.28) | -3.2(-3.36,-3.04) |
| Latin America | Panama | 962.61(488.88,1603.01) | 23.14(11.75,38.53) | 23.03(11.71,38.43) | -1.99(-2.47,-1.51) |
| Latin America | Uruguay | 10131.71(7833.54,12692.83) | 294.86(227.98,369.39) | 185.82(144.32,232.67) | -3.32(-3.52,-3.13) |
| Latin America | Nicaragua | 1412.22(492.53,2609.39) | 21.69(7.57,40.08) | 34.9(12.08,64.36) | -0.68(-1.06,-0.29) |
| Latin America | Ecuador | 17376.14(12586.49,23008.05) | 98.79(71.56,130.81) | 116.96(84.9,153.84) | -1.68(-1.83,-1.53) |
| Latin America | Brazil | 173213.04(115480.69,240857.31) | 79.95(53.3,111.17) | 73.28(48.46,102.01) | -3.78(-3.84,-3.71) |
| Latin America | Colombia | 36687.63(24903.14,50259.51) | 76.79(52.12,105.2) | 68.92(46.67,94.68) | -3.65(-3.75,-3.54) |
| Latin America | Cuba | 4579.86(1711.84,9193.96) | 40.32(15.07,80.94) | 24.04(8.95,48.58) | -2.37(-2.62,-2.11) |
| Latin America | Belize | 74.04(32.5,130.44) | 18.06(7.93,31.81) | 26.44(11.7,46.5) | -2.41(-3.94,-0.85) |
| Latin America | Jamaica | 254.25(6.28,965.58) | 9.05(0.22,34.35) | 8.39(0.2,31.98) | -1.83(-2.64,-1.02) |
| Latin America | Chile | 42973.96(31003.28,57261.98) | 236.14(170.36,314.65) | 181.06(130.88,240.97) | -2.23(-2.45,-2.01) |
| Latin America | El Salvador | 737.06(231.77,1510.55) | 11.78(3.7,24.15) | 12.15(3.81,24.94) | -2.63(-3.03,-2.23) |
| Latin America | Suriname | 111.37(18.47,297.51) | 19.34(3.21,51.66) | 18.47(3.06,49.17) | -0.57(-2.16,1.05) |
| Latin America | Dominican Republic | 10349.86(5527.09,16591.7) | 95.11(50.79,152.47) | 110.23(59.22,176.7) | 1.18(1.04,1.33) |
| Latin America | Mexico | 169442.54(128870.1,218197.42) | 135.62(103.15,174.64) | 146.93(111.91,189.27) | -1.15(-1.24,-1.06) |
| Latin America | Haiti | 5092.75(1304.18,11460.75) | 41.06(10.52,92.41) | 71.58(18.81,160.43) | -0.75(-0.94,-0.56) |
| Latin America | Dominica | 26.69(2.4,79.3) | 38.87(3.5,115.47) | 29.74(2.66,88.66) | -2.02(-5.96,2.08) |
| Latin America | Costa Rica | 3149.66(2036.8,4562.83) | 66.78(43.18,96.74) | 61.16(39.51,88.56) | -2.05(-2.33,-1.77) |
| Latin America | Guyana | 537.65(223.44,973.66) | 69.76(28.99,126.33) | 85.06(35.54,153.08) | -1.66(-2.21,-1.12) |
| Latin America | Honduras | 7065.25(3149.62,12722.08) | 71.99(32.09,129.63) | 120.67(55.22,213.35) | -0.89(-1.14,-0.65) |
| Latin America | Argentina | 116892.07(91726.15,144780.39) | 259.1(203.32,320.91) | 218.55(171.35,270.9) | -3.53(-3.66,-3.4) |
| Latin America | Peru | 31049.17(20882.75,44350.7) | 91.33(61.43,130.46) | 94.73(63.61,135.68) | -3(-3.12,-2.89) |
| Latin America | Paraguay | 6050.55(3086.95,9902.28) | 87.3(44.54,142.88) | 108.75(55.59,177.81) | -1.79(-2,-1.58) |
| Latin America | Barbados | 49.09(4.64,135.35) | 16.49(1.56,45.45) | 10.15(0.96,27.91) | -3.32(-6.55,0.02) |
| Latin America | Puerto Rico | 980.02(196.8,2718.06) | 27.83(5.59,77.19) | 14.29(2.9,38.83) | -3.62(-4.29,-2.95) |
| Middle East | Egypt | 372601.2(196987.05,583366.19) | 376.1(198.84,588.85) | 584.75(314.3,906.05) | -0.88(-1.02,-0.74) |
| Middle East | Iran (Islamic Republic of) | 252102.91(203097.71,301365.85) | 299.06(240.93,357.5) | 359(289.47,429.82) | -3.27(-3.38,-3.16) |
| Middle East | Palestine | 9389.94(5466.17,13600.79) | 189.44(110.28,274.4) | 428.19(249.06,620.52) | -2.86(-3.02,-2.7) |
| Middle East | Yemen | 45691.64(21200.19,70589.64) | 145.04(67.3,224.07) | 334.59(158.14,510.56) | -1.54(-1.62,-1.47) |
| Middle East | Djibouti | 643.63(19.83,1237.88) | 53.51(1.65,102.92) | 109.82(3.47,205.8) | -0.35(-0.88,0.19) |
| Middle East | Kuwait | 6119.75(1020.36,10827.33) | 138.25(23.05,244.6) | 201.36(37.75,357.57) | -2.02(-2.21,-1.83) |
| Middle East | Jordan | 19109.86(12408.64,27051.97) | 164.22(106.63,232.47) | 311.15(204.53,439.7) | -3.79(-3.97,-3.61) |
| Middle East | Libya | 16144.83(7933.73,25158.59) | 239.7(117.79,373.52) | 305.67(153.72,476.19) | -0.58(-0.78,-0.37) |
| Middle East | Oman | 5327.5(230.6,10409.69) | 116.22(5.03,227.09) | 348.96(14.85,672.05) | -3.45(-3.88,-3.02) |
| Middle East | Morocco | 184471.68(134303.98,239173.4) | 513.1(373.56,665.25) | 618.95(456.06,787.07) | -1.8(-1.95,-1.64) |
| Middle East | Qatar | 1689.76(0,3425.6) | 58.99(0,119.59) | 249.38(0,491.92) | -4.85(-5.18,-4.52) |
| Middle East | Tunisia | 46899.34(29140.78,69179.49) | 405.3(251.83,597.84) | 388.01(240.72,569.9) | -1.57(-1.65,-1.49) |
| Middle East | Iraq | 115575(70396.94,165175.28) | 274.4(167.14,392.16) | 512.94(314.82,722.17) | -2.35(-2.46,-2.23) |
| Middle East | Israel | 8701.02(4696.48,13290.2) | 93.46(50.45,142.76) | 73.35(39.96,111.65) | -6.43(-6.7,-6.15) |
| Middle East | Lebanon | 23647.56(14899.37,32624.89) | 456.78(287.8,630.18) | 455.34(287.73,626.7) | -2.05(-2.19,-1.92) |
| Middle East | Malta | 1328.13(489.53,2330.83) | 302.38(111.45,530.67) | 143.02(52.44,250.18) | -3.34(-3.95,-2.72) |
| Middle East | Syrian Arab Republic | 81157.92(53308.25,118276.71) | 560.05(367.87,816.19) | 689.94(460,988.91) | -2.57(-2.68,-2.45) |
| Middle East | United Arab Emirates | 15868.95(0,34424.25) | 171.71(0,372.49) | 313.5(-0.81,658.99) | -2.44(-2.73,-2.15) |
| Middle East | Bahrain | 1328.21(312.12,2322.9) | 92.06(21.63,161.01) | 151(36.77,263.73) | -5.68(-6,-5.37) |
| Middle East | Algeria | 111163.49(65591.53,159620.93) | 265.64(156.74,381.44) | 361.57(214.77,514.12) | -3.22(-3.33,-3.12) |
| Middle East | Saudi Arabia | 65771.04(30576.57,101384.16) | 184.07(85.57,283.74) | 318.18(149.7,483.77) | -1.05(-1.19,-0.91) |
| North America | United States of America | 1023065.75(822912.17,1230926.36) | 311.93(250.9,375.31) | 188.31(151.97,225.71) | -2.36(-2.55,-2.17) |
| North America | Bermuda | 116.54(0,273.67) | 182.01(0,427.41) | 89.59(0,210.7) | -4.49(-6.7,-2.23) |
| North America | Canada | 109120.6(35846.47,183274.21) | 298.8(98.16,501.85) | 158.04(51.8,264.3) | -3.41(-3.61,-3.2) |
| South Asia | Bangladesh | 159224.2(69837,262907.04) | 99.98(43.85,165.08) | 122.11(53.37,201.43) | -1.04(-1.28,-0.8) |
| South Asia | Pakistan | 490594.69(229938.68,759500.67) | 218.95(102.62,338.97) | 408.53(192.14,625.67) | 0.35(0.29,0.42) |
| South Asia | Afghanistan | 136284.12(94669.56,188164.1) | 356.04(247.32,491.58) | 964.41(681.45,1290.24) | -1.81(-1.87,-1.74) |
| South Asia | Nepal | 78610.8(54589.31,108211.66) | 258.45(179.47,355.77) | 357.72(251.03,487.6) | -1.23(-1.32,-1.15) |
| South Asia | Bhutan | 2185.67(1446.47,3105.19) | 289.78(191.78,411.69) | 393.05(263.06,549.1) | -1.76(-2.06,-1.46) |
| South Asia | Sri Lanka | 7231(3033.59,11800.53) | 33.09(13.88,54) | 29.67(12.38,48.31) | -2.57(-2.75,-2.4) |
| South Asia | Maldives | 0.37(-0.13,3.05) | 0.07(-0.03,0.61) | 0.12(-0.05,0.99) | -6.28(-8.48,-4.02) |
| South Asia | India | 1756579.93(859442.5,2665341.98) | 126.31(61.8,191.65) | 151.07(74.35,228.65) | -0.89(-1.05,-0.72) |
| Sub Saharan Africa | Congo | 1699.3(367.14,3821.52) | 32.27(6.97,72.57) | 66.69(14.7,144.33) | -3.01(-3.29,-2.72) |
| Sub Saharan Africa | Cote d'Ivoire | 2202.94(802.64,4477.1) | 8.42(3.07,17.11) | 20.32(7.52,40.73) | -2.31(-2.54,-2.07) |
| Sub Saharan Africa | Democratic Republic of the Congo | 50828.9(29443.96,79087.8) | 57.98(33.58,90.21) | 145.23(84.55,222.73) | -1.11(-1.16,-1.05) |
| Sub Saharan Africa | Gambia | 452.39(66.51,778.4) | 20.14(2.96,34.66) | 47.12(7.1,79.54) | 0.39(-0.46,1.25) |
| Sub Saharan Africa | Sao Tome and Principe | 21.76(-0.56,86.58) | 10.6(-0.27,42.16) | 21.32(-0.17,82.66) | -1.27(-4,1.54) |
| Sub Saharan Africa | United Republic of Tanzania | 37077.42(20826.97,59934.94) | 65.35(36.71,105.64) | 153.65(87.21,242.1) | -1.4(-1.48,-1.32) |
| Sub Saharan Africa | Zambia | 15205(5994.24,29192.18) | 83.37(32.87,160.07) | 223.61(89.2,418.06) | -0.19(-0.29,-0.08) |
| Sub Saharan Africa | Botswana | 3121.93(1286.43,5876.59) | 133.49(55.01,251.27) | 231.98(94.98,428.4) | -0.64(-0.89,-0.39) |
| Sub Saharan Africa | Comoros | 152.56(5.19,486.15) | 21.36(0.73,68.05) | 31.92(1.16,101.32) | -2.25(-3.22,-1.28) |
| Sub Saharan Africa | Guinea-Bissau | 358.03(9.03,692.93) | 18.83(0.48,36.45) | 46.75(1.17,88.49) | 0.36(-0.16,0.88) |
| Sub Saharan Africa | Ethiopia | 67218.03(49346.73,90553.36) | 62.48(45.87,84.16) | 165.25(121.98,219.22) | -3.93(-4.04,-3.82) |
| Sub Saharan Africa | South Sudan | 857.8(249.81,1544.9) | 9.24(2.69,16.64) | 22.8(6.72,40.28) | -3.63(-3.95,-3.31) |
| Sub Saharan Africa | Lesotho | 8339.21(5300.46,11915.36) | 398.7(253.42,569.68) | 674.48(436.77,954.02) | 2.18(1.98,2.37) |
| Sub Saharan Africa | Niger | 14490.72(717.29,29474.97) | 62.2(3.08,126.53) | 179.33(11.53,351.18) | -1.5(-1.63,-1.38) |
| Sub Saharan Africa | Benin | 1323.77(44.73,2580.53) | 10.45(0.35,20.37) | 26.76(0.91,50.45) | -2.74(-3.1,-2.39) |
| Sub Saharan Africa | Eswatini | 2164.17(1349.35,3330.01) | 189.49(118.15,291.57) | 381.82(241.95,577.53) | 0.79(0.48,1.1) |
| Sub Saharan Africa | Senegal | 3615.01(282.43,6508.57) | 23.89(1.87,43.01) | 48.82(3.91,86.75) | 0.38(0.16,0.61) |
| Sub Saharan Africa | Cabo Verde | 644.68(139.89,1364.56) | 114.39(24.82,242.13) | 152.56(33.42,320.17) | -0.84(-1.43,-0.24) |
| Sub Saharan Africa | Seychelles | 9.52(0.17,33.82) | 9.32(0.17,33.11) | 8.76(0.15,31.33) | -3.03(-6.17,0.22) |
| Sub Saharan Africa | Chad | 10402.81(2382.14,19222.53) | 63.44(14.53,117.22) | 174.39(44.42,318.04) | -0.66(-0.79,-0.52) |
| Sub Saharan Africa | Uganda | 15170.1(6903.37,26254.75) | 36.89(16.79,63.85) | 107.18(50.11,182.63) | -1.4(-1.53,-1.26) |
| Sub Saharan Africa | Kenya | 36897.36(25446.26,51104.1) | 73.46(50.66,101.74) | 172.08(120.74,235.79) | 0.01(-0.14,0.16) |
| Sub Saharan Africa | South Africa | 107840.77(90616.16,127573.39) | 194(163.01,229.5) | 249.97(209.91,294.49) | -2.31(-2.79,-1.84) |
| Sub Saharan Africa | Ghana | 2616.37(424.35,5231.46) | 8.3(1.35,16.59) | 16.54(2.74,32.48) | -3.4(-3.61,-3.19) |
| Sub Saharan Africa | Mauritius | 1482.68(0,3970.46) | 116.14(0,311) | 87.79(0,234.05) | -4.8(-5.15,-4.44) |
| Sub Saharan Africa | Liberia | 530.11(64.14,1592.52) | 11.07(1.34,33.25) | 25.4(3.03,74.63) | -1.74(-2.27,-1.21) |
| Sub Saharan Africa | Sierra Leone | 898.28(214.59,2186.6) | 10.84(2.59,26.39) | 24.01(6.09,57.53) | -0.9(-1.27,-0.52) |
| Sub Saharan Africa | Mauritania | 3394.81(362.41,6563.83) | 84.57(9.03,163.51) | 167.05(19.54,320.48) | -2.17(-2.42,-1.91) |
| Sub Saharan Africa | Somalia | 5518.78(2820.2,8835.09) | 27.13(13.86,43.43) | 79.12(39.87,123.9) | -0.69(-0.86,-0.53) |
| Sub Saharan Africa | Cameroon | 10044.53(4243.25,18518.18) | 34.52(14.58,63.63) | 82.13(35.51,149.87) | -0.58(-0.71,-0.45) |
| Sub Saharan Africa | Burundi | 13251.36(6287.12,22027.63) | 111.04(52.68,184.57) | 284.89(139.9,468.39) | -2.36(-2.46,-2.26) |
| Sub Saharan Africa | Eritrea | 3315.08(1486.56,5313.84) | 49.4(22.15,79.18) | 120.99(56.5,190.95) | -2.43(-2.62,-2.24) |
| Sub Saharan Africa | Malawi | 13993.14(8036.79,21932.18) | 75.88(43.58,118.92) | 189.19(110.37,292.72) | -0.83(-0.94,-0.72) |
| Sub Saharan Africa | Namibia | 3119.45(1723.77,4948.17) | 129.81(71.73,205.91) | 230.3(128.33,363.75) | -1.46(-1.73,-1.19) |
| Sub Saharan Africa | Rwanda | 15247.88(9330.8,23105.18) | 120.17(73.54,182.1) | 264.87(164.71,392.13) | -4.38(-4.51,-4.25) |
| Sub Saharan Africa | Sudan | 61663.03(23494.91,102856.53) | 151.1(57.57,252.05) | 319.72(121.76,527.38) | -2.55(-2.61,-2.49) |
| Sub Saharan Africa | Togo | 691.61(186.78,1345.05) | 8.73(2.36,16.98) | 18.78(5.26,35.42) | -2.94(-3.36,-2.51) |
| Sub Saharan Africa | Guinea | 2384.83(984.96,4254.18) | 18.86(7.79,33.65) | 41.87(17.94,72.96) | 0.22(-0.06,0.5) |
| Sub Saharan Africa | Central African Republic | 1462.36(600.87,2726.17) | 27.59(11.34,51.44) | 64.85(27.49,118.89) | -1.45(-1.74,-1.17) |
| Sub Saharan Africa | Mali | 15794.43(-1109.16,34033.3) | 72.06(-5.06,155.28) | 178.98(-3.35,378.69) | -1.34(-1.45,-1.22) |
| Sub Saharan Africa | Madagascar | 31656.84(19734.96,47176.67) | 118.61(73.94,176.76) | 272.44(170.6,399.23) | -1.19(-1.32,-1.05) |
| Sub Saharan Africa | Zimbabwe | 16812.92(9939.62,26496.29) | 112.01(66.22,176.51) | 248.78(148,385.92) | 2.33(2.15,2.51) |
| Sub Saharan Africa | Gabon | 503.19(127.62,1070.07) | 28.75(7.29,61.15) | 50.03(12.93,105.21) | -3(-3.53,-2.47) |
| Sub Saharan Africa | Equatorial Guinea | 302.44(46.79,775.57) | 21.3(3.3,54.62) | 66.18(11.09,164.93) | -5.16(-5.81,-4.5) |
| Sub Saharan Africa | Mozambique | 17749.82(7788.3,30665.44) | 60.11(26.38,103.85) | 160.69(73.09,272.98) | 1.83(1.72,1.95) |
| Sub Saharan Africa | Burkina Faso | 6527.97(6.54,12167.05) | 28.77(0.03,53.62) | 69.66(0.07,127.39) | -0.79(-0.97,-0.61) |
| Sub Saharan Africa | Nigeria | 19203.18(2372.17,44681.74) | 8.94(1.1,20.8) | 21.22(2.72,48.13) | -3.82(-3.94,-3.69) |
| Sub Saharan Africa | Angola | 18417.7(10345.25,29592.14) | 61.11(34.33,98.19) | 167.36(94.92,266.23) | -1.77(-1.86,-1.67) |
